# Genetic and clinical landscape of Chinese frontotemporal dementia: dominance of *TBK1* and *OPTN* mutations

**DOI:** 10.1186/s13195-024-01493-w

**Published:** 2024-06-13

**Authors:** Haitian Nan, Yeon-Jeong Kim, Min Chu, Dan Li, Jieying Li, Deming Jiang, Yiming Wu, Toshihisa Ohtsuka, Liyong Wu

**Affiliations:** 1 Department of Neurology, Xuanwu Hospital, Capital Medical University, 45 Changchun Street, Beijing, 100053 China; 2 Department of Biochemistry, Graduate School of Medical Sciences, University of Yamanashi, Yamanashi, 409-3898 Japan; 3 Sichuan Provincial Center for Mental Health, Sichuan Academy of Medical Science & Sichuan Provincial People’s Hospital, Chengdu, 610072 China; 4 The Experimental High School Attached to Beijing Normal University, Beijing, 100032 China

**Keywords:** Frontotemporal dementia, Genetic spectrum, *TBK1*, *OPTN*, Autophagy

## Abstract

**Background:**

Our study aims to evaluate the genetic and phenotypic spectrum of Frontotemporal dementia (FTD) gene variant carriers in Chinese populations, investigate mutation frequencies, and assess the functional properties of *TBK1* and *OPTN* variants.

**Methods:**

Clinically diagnosed FTD patients underwent genetic analysis through exome sequencing, repeat-primed polymerase chain reaction, and Sanger sequencing. *TBK1* and *OPTN* variants were biologically characterized in vitro using immunofluorescence, immunoprecipitation, and immunoblotting analysis. The frequencies of genes implicated in FTD in China were analyzed through a literature review and meta-analysis.

**Results:**

Of the 261 Chinese FTD patients, 61 (23.4%) carried potential causative variants in FTD-related genes, including *MAPT* (*n* = 17), *TBK1* (*n* = 7), *OPTN* (*n* = 6), *GRN* (*n* = 6), *ANXA11* (*n* = 4), *CHMP2B* (*n* = 3), *C9orf72* GGGGCC repeats (*n* = 2), *CYLD* (*n* = 2), *PRNP* (*n* = 2), *SQSTM1* (*n* = 2), *TARDBP* (*n* = 2), *VCP* (*n* = 1), *CCNF* (*n* = 1), *CHCHD10* (*n* = 1), *SIGMAR1* (*n* = 1), *CHCHD2* (*n* = 1), *FUS* (*n* = 1), *TMEM106B* (*n* = 1), and *UBQLN2* (*n* = 1). 29 variants can be considered novel, including the *MAPT* p.D54N, p.E342K, p.R221P, p.T263I, *TBK1* p.E696G, p.I37T, p.E232Q, p.S398F, p.T78A, p.Q150P, p.W259fs, *OPTN* p.R144G, p.F475V, *GRN* p.V473fs, p.C307fs, p.R101fs, *CHMP2B* p.K6N, p.R186Q, *ANXA11* p.Q155*, *CYLD* p.T157I, *SQSTM1* p.S403A, *UBQLN2* p.P509H, *CCNF* p.S160N, *CHCHD10* p.A8T, *SIGMAR1* p.S117L, *CHCHD2* p.P53fs, *FUS* p.S235G & p.S236G, and *TMEM106B* p.L144V variants. Patients with *TBK1* and *OPTN* variants presented with heterogeneous clinical phenotypes. Functional analysis demonstrated that TBK1 I37T and E232Q mutants showed decreased autophosphorylation, and the OPTN phosphorylation was reduced by the TBK1 I37T mutant. The OPTN-TBK1 complex formation was enhanced by the TBK1 E696G mutant, while OPTN R144G and F475V mutants exhibited reduced recruitment to autophagosomes compared to the wild-type. The overall frequency of *TBK1* and *OPTN* in Chinese FTD patients was 2.0% and 0.3%, respectively.

**Conclusions:**

Our study demonstrates the extensive genetic and phenotypic heterogeneity of Chinese FTD patients. *TBK1* mutations are the second most frequent cause of clinical FTD after *MAPT* in the Chinese.

**Supplementary Information:**

The online version contains supplementary material available at 10.1186/s13195-024-01493-w.

## Introduction

Frontotemporal dementia (FTD) is the second most common cause of early-onset dementia [1]. FTD is clinically categorized into behavioral variant FTD (bv-FTD) and primary progressive aphasia (PPA), the latter consisting of nonfluent/agrammatic variant primary progressive aphasia (nvPPA) and semantic variant primary progressive aphasia (svPPA) subtypes based on clinical features [2]. Moreover, FTD occasionally overlaps with motor neuron disease such as amyotrophic lateral sclerosis (ALS), or atypical parkinsonian syndromes such as progressive supranuclear palsy (PSP) and corticobasal syndrome (CBS) [1].

**Fig. 1:**
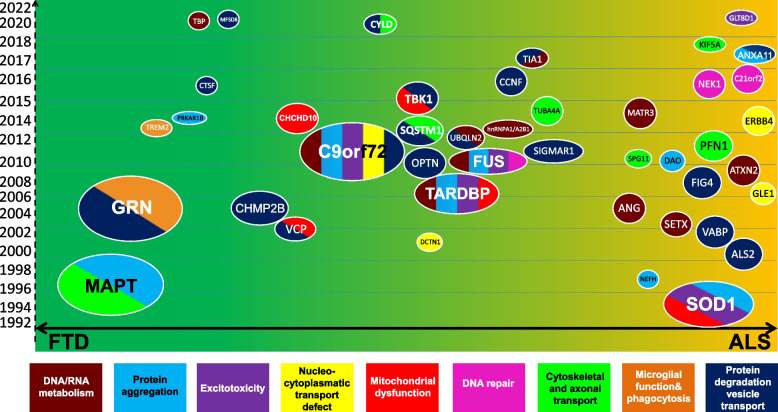
The landscape of disease-causing genes of FTD and ALS. Some FTD-causing genes overlap with ALS genes. The position of a gene depends on when it was discovered and how specifically it is associated with the clinical phenotypes of ALS (yellow) and FTD (green). The timelines of the genes discovered are shown in the Y axis. Genes are plotted according to their hypothesized mechanism in relation to disease. The size of a gene icon depends on the frequency of its occurrence and its clinical and functional significance

FTD frequently has a strong genetic component contributing to its pathogenesis. In Caucasian populations, around 30% to 50% of patients with FTD have a family history [3], of which mutations involving *GRN*, *MAPT*, and *C9orf72* (chromosome 9 open reading frame 72) account for 60% of all inherited FTD [4]. Recently, it has become increasingly apparent that ALS and FTD share significant genetic overlap, and mutations in an increasing number of genes have been associated with autosomal dominant FTD (Fig. 1) [4–6]. Recent studies have identified *TBK1* as probably the fourth most common genetic cause overall of FTD, accounting for between 1 and 2% of all cases [7, 8].

The genetic landscape of FTD is different between Asians and Caucasians. Although *C9orf72* has been reported as the most common pathogenic gene in Western countries [9], *MAPT* is thought to be the most common pathogenic gene for FTD in China [10]. Moreover, the proportion of Asian patients with FTD who have a positive family history ranges from 9.5% to 20% [11]. The majority of Asian FTD patients are sporadic, and comprehensive analyses of mutations in sporadic FTD patients may provide a better understanding of the genetic causes of FTD in Asian populations.

To date, limited data are available on the genetic spectrum of Chinese FTD patients [8, 10], and there is a lack of functional studies to verify the pathogenicity of the disease-causing mutations. Herein, we aim to expand the genetic diversity of Chinese FTD patients by analyzing their genetic spectrum and phenotypic traits. We investigate both familial and sporadic FTD patients, with a focus on the latter due to their relatively higher prevalence in Asian populations. Additionally, we conducted functional studies on mutations in *TBK1* and *OPTN* to elucidate their underlying mechanisms. Lastly, the overall frequency of *TBK1* and *OPTN* variants in Chinese FTD patients was calculated using meta-analysis.

## Material and methods

### Participants

This study included 261 unrelated participants of Chinese Han ancestry, including 32 familial FTD index patients from 32 pedigrees and 229 sporadic FTD patients. An FTD database was established at the Department of Neurology of Xuanwu Hospital, China, which included 229 sporadic FTD patients and 49 familial FTD patients from 32 pedigrees who were consecutively recruited between July 1, 2014, and March 31, 2024. Family history was investigated for up to 3 sequential generations for FTD patients. We defined sporadic FTD as patients with no known family history of neuropsychiatric disorders, including dementia, ALS, Parkinson's syndromes, psychosis, depression, and suicide. We defined FTD as familial when at least two individuals within a 3rd-degree relationship were affected with FTD or similar neuropsychiatric disorders. Within one month of recruitment, all patients underwent comprehensive evaluations, including clinical interviews, physical examinations, neuropsychological assessments, genetic testing, and neuroimaging studies including ^18^F-fluorodeoxyglucose positron emission tomography (^18^F-FDG PET) or magnetic resonance imaging examinations (MRI).

This study was conducted as part of the Chinese Frontotemporal Dementia Genomics Study (CHIFGENS). Thus, all participants were registered in the CHIFGENS registry. The study was approved by the Ethics Committees of the Xuanwu Hospital of Capital Medical University, China (protocol code 2,020,026), and was conducted by the principles stated in the Declaration of Helsinki. Written informed consent was obtained from each patient or their guardian.

### DNA isolation, PRNP octapeptide repeat analysis, and C9orf72 genotyping

Genomic DNA was extracted from peripheral blood lymphocytes following a standard protocol. All DNA samples were normalized to 50–100 ng/μl. The presence of the insertion or deletion of octapeptide repeats in *PRNP* was verified by nested polymerase chain reaction (PCR) and agarose electrophoresis as previously described [12]. The hexanucleotide repeat expansions in *C9orf72* were also detected by adopting the methods previously described [13].

### Whole-exome sequencing (WES) study

To comprehensively investigate the potential genetic cause of these patients, we performed WES of genomic DNA from 261 FTD patients. We summarized FTD and other dementia-related and susceptible genes using the Online Mendelian Inheritance in Man (OMIM) and PubMed database (Table S1). Exome capture was performed with a SureSelect Human All Exon V6 + UTR (89 Mb) Kit (Agilent Technologies, Santa Clara, CA, USA). Paired-end sequencing was carried out on a HiSeq2500 (Illumina, San Diego, CA, USA) using a HiSeq SBS Kit V4 (Illumina), which generated 100-bp reads. The average and minimum sequencing depths were 205 × and 10 × , respectively. The reference databases utilized included hg38 (GRCh38) (http://genome.ucsc.edu), HGMD (https://portal.biobase-international.com), gnomAD (http://gnomad.broadinstitute.org), ClinVar (https://www.ncbi.nlm.nih.gov/clinvar/), and dbSNP (https:// www. ncbi. nlm. nih. gov/SNP). WES data were analyzed for single-nucleotide variants (SNVs) and insertion-deletions (InDels) in dementia-related genes. The significant results were comprehensively evaluated in aspects including minor allele frequency, conservation, predicted pathogenicity, disease association, and confirmation with Sanger sequencing. All heterozygous variants with a minor allele frequency < 0.1%, as well as homozygous and potentially compound heterozygous variants, were considered.

Cases were considered to have a definite genetic diagnosis if a variant was classified as pathogenic or likely pathogenic according to the American College of Medical Genetics and Genomics (ACMG) guidelines [14]. For assessment of the *ApoE* status, the three alleles *ApoE2*, *ApoE3,* and *ApoE4* were determined according to the presence of variants rs7412 and rs429358 in the WES data. The distribution of *ApoE* alleles in our FTD cohort was compared with the normal Chinese population in a previous study [15].

### Plasmids and constructs

The coding regions of human *TBK1* and *OPTN* cDNAs were amplified using PCR, and then subcloned into pmCherry-C1 (Clontech) and pUY-3 × Flag (home-made) vectors. TBK1 and OPTN amino acid substitutions were introduced into Flag-TBK1 wild-type or pmCherry-OPTN wild-type using PCR-based site-directed mutagenesis. We verified the complete nucleotide sequences of the expression plasmids.

### Cell culture and transfection

HEK293T cells were maintained in Dulbecco's Modified Eagle's Medium supplemented with 10% fetal bovine serum and a non-essential amino acids solution (Thermo Fisher Scientific). DNA transfection was performed by the polyethyleneimine-based method [16] using PEI MAX reagents (Polysciences). After 48 h of transfection, cells were harvested and lysed in lysis buffer (20 mM Tris–HCl, 150 mM NaCl, 5 mM MgCl_2,_ 1% Triton X-100) supplemented with cOmplete™ protease inhibitor cocktail (Roche) and phosphatase inhibitor cocktail, PhosSTOP.

### Immunoprecipitation and western blotting

The cell lysate underwent centrifugation at 20,000 × g for 10 min at 4 °C, after which the supernatant was collected. Protein samples were mixed with anti-DDDDK antibody-coupled magnetic beads (MBL) for 30 min, and then the immunoprecipitates were washed extensively with the lysis buffer. Proteins were eluted from the beads in the Laemmli sample buffer by boiling and then subjected to SDS-PAGE. The separated proteins were transferred to PVDF membranes (Millipore). Anti-DDDDK-tag (MBL) and anti-RFP (MBL) antibodies were used for western blotting. Autophosphorylation of TBK1 was detected with a phosphospecific antibody (pS172: no. 5483; Cell Signaling Technology). The phosphorylation of S177 in OPTN was detected using a phosphospecific antibody (pS177: no 31304). The antibody-targeted proteins were detected using Immobilon Forte chemiluminescence HRP substrate (Millipore) and imaged using an ImageQuant LAS 4000 system (Cytiva).

### Immunofluorescence and confocal laser scanning microscopy

Neuro2A (mouse neuroblastoma) cell lines were maintained in DMEM (Gibco) supplemented with 10% fetal bovine serum and 1% penicillin–streptomycin (Gibco) and plated on coverslips for immunofluorescence staining. Cells were transfected with pmCherry-OPTN wild-type and mutants by using PEI MAX transfection reagent (Polysciences). After 24 h of transfection, cells were treated with 100 nM Bafilomycin A1 (Sigma) for 3 h in Hanks’ balanced salt solution with magnesium and calcium. And then, cells were fixed in 4% paraformaldehyde and permeabilized with 0.3% Triton X-100. Cells were subsequently labeled with anti-LC3A/B antibody (Cell signaling technology), and then subsequently stained with Alex Fluor 488 labeled anti-rabbit IgG antibody. The images were captured as a single confocal plane using the FV1000 system (Olympus) using a × 100 oil immersion objective lens.

### Image analysis and statistics

We performed image analysis to determine the co-localization between OPTN and LC3A/B in 30 randomly selected fields of images. ImageJ software (http://rsbweb.nih.gov/ij/) was used for the analysis, and the Coloc2 plugin was utilized to measure Pearson's *R* values. Statistical analysis was conducted using GraphPad Prism software through a one-way ANOVA.

### Literature review and *meta*-analysis

First, we searched PubMed, Embase, and Web of Science for relevant literature from inception until March 2024 and conducted a meta-analysis of the frequency of variants. We searched with disease-related terms of frontotemporal dementia, frontotemporal lobar degeneration, primary progressive aphasia, progressive nonfluent aphasia, semantic dementia, and gene-related terms of polymorphism, genetics, or gene, and geographically relevant terms of China, Han, and Chinese by theme in the title and abstract, and there were 174 results. We next searched for FTD cohort studies performed in Chinese FTD patients with each cohort of more than 10 FTD patients. Twenty results remained that were closely related. Finally, we summarized the characteristics of each FTD gene. The effect sizes and corresponding 95% confidence interval (CI) were calculated for each study. Statistical heterogeneity among studies was evaluated using Cochrane’s Q test (significance set at *p* < 0.10) and *I*^*2*^ statistics. If *I*^*2*^ < 50%, then the studies were considered to be homogeneous, and the combined mutation frequencies were calculated using a common effect model. Otherwise,* I*^*2*^ > 50% or* p* for Q test < 0.10 indicates substantial heterogeneity across studies.

Statistical analysis was performed using the statistical software SPSS (IBM SPSS Statistics for Windows, version 26, IBM Corporation, Inc., Chicago, IL, USA, 2019). We performed a meta-analysis of variant frequency using R software (version 4.1.2) and the “meta” package (version 7.0.0) to calculate effect sizes and 95% confidence intervals (CIs) for each previously reported study.

## Results

### Demographic features and clinical diagnosis of FTD patients

**Fig. 2:**
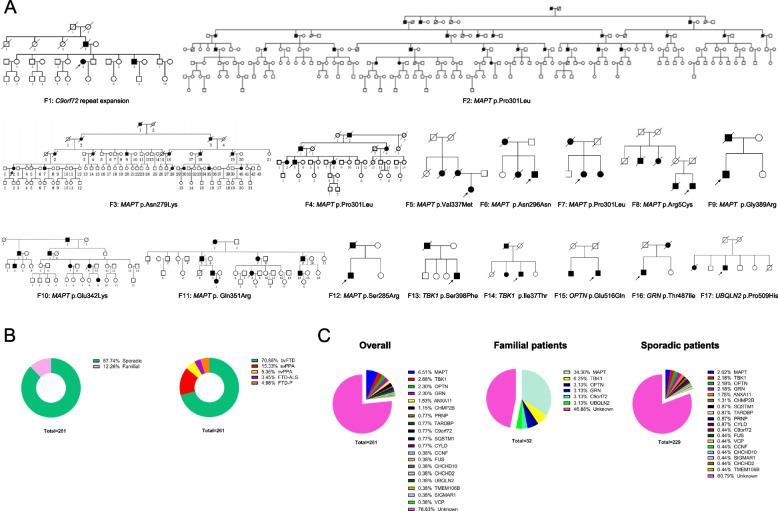
Genetic analysis of the FTD participants. **A** Pedigree charts of the 17 familial FTD patients in our cohort. **B** The proportion of familial and sporadic cases and the phenotype spectrum of the FTD participants in our cohort. **C** The genetic spectrum of the FTD participants in our cohort

The demographic features of the 261 FTD patients are shown in Table 1. A total of 32 patients (12.3%) had a positive family history in first-degree or second-degree relatives (Fig. 2A, B). 53.1% (17/32) of individuals with a positive family history and 19.2% (44/229) of sporadic patients could be genetically diagnosed.

**Table 1:** Demographic data of FTD patients

	Overall	Sporadic FTD	Familial FTD	bvFTD	svPPA	nvPPA	FTD-ALS	FTD-P
Cases, *n*	261	229 (87.7%)	32 (12.3%)	185 (70.9%)	40(15.3%)	14(5.4%)	9(3.5%)	13(5.0%)
Female, *n* (%)	144 (55.2%)	129 (56.3%)	15 (46.9%)	103 (55.7%)	21 (52.1%)	8 (57.1%)	5 (55.6%)	7 (53.8%)
Age (years)	60.2 ± 10.6	61.3 ± 10.4	54.5 ± 3.0	61.7 ± 8.9	59.3 ± 4.5	59.6 ± 4.1	62.8 ± 2.2	63.4 ± 2.7
AAO (years)	57.7 ± 10.9	58.8 ± 10.1	52.6 ± 3.0	58.8 ± 8.6	57.2 ± 4.5	56.6 ± 4.4	61.0 ± 2.4	59.5 ± 2.8
Early-onset n (%)	237 (90.8%)	207 (90.4%)	30 (93.8%)	169 (91.4%)	37 (92.5%)	14 (100%)	8 (88.9%)	9 (69.2%)
Minimum disease duration^a^ (years)	2.6 ± 1.8	2.5 ± 1.8	2.0 ± 1.7	2.8 ± 1.9	2.1 ± 1.6	3.1 ± 2.0	1.9 ± 1.8	3.9 ± 2.2
MMSE score	17.8 ± 6.9	18.0 ± 6.6	16.8 ± 6.3	17.6 ± 6.7	15.6 ± 5.2	15.4 ± 4.7	20.2 ± 3.6	18.4 ± 2.3
MoCA score	11.5 ± 6.4	12.2 ± 6.1	10.5 ± 5.7	11.0 ± 6.2	9.9 ± 5.6	10.5 ± 4.9	15.6 ± 4.1	12.4 ± 1.8
Genetically diagnosed	61 (23.4%)	44 (19.2%)	17 (53.1%)	42 (22.7%)	9 (22.5%)	0	4 (44.4%)	6 (46.2%)

*FTD* frontotemporal dementia, *bvFTD* behavioral variant of FTD, *nvPPA* nonfluent/agrammatic variant primary progressive aphasia, *svPPA* semantic variant primary progressive aphasia, *ALS* amyotrophic lateral sclerosis, *FTD-P* FTD-parkinsonism overlap, *AAO* age at onset

^a^*Minimum disease duration is the span of time from the onset of the disease to the last follow-up in most cases of our cohort*

All patients with FTD met the consensus criteria for FTD spectrum [17]: 185 for bv-FTD [18], 14 for nvPPA [2], 40 for svPPA [2], 9 for FTD combined with ALS [19], and 13 for FTD-parkinsonism overlap (FTD-P) [20] (Fig. 2B).

### Genetic findings

A total of 261 individuals were screened for dementia-related causing and susceptible genes. We were able to establish a genetic diagnosis in 17 of 32 familial cases (Fig. 2A). Family 1 had a repeat expansion in *C9orf72*. Family 2, Family 4, and Family 7 had the p.P301L mutation in *MAPT*. Family 3, Family 5, Family 6, Family 8, Family 9, Family 10, Family 11, and Family 12 had p.N279K, p.V337M, p.N296N, p.R5C, p.G389R, p.E342K, p.Q351R, and p.S285R mutations in *MAPT* respectively. Family 13 and Family 14 had p.S398F and p.I37T mutations in *TBK1*, respectively. One family each was found to carry a variant in *OPTN*, *GRN*, and *UBQLN2*, respectively. Of the sporadic cases, repeat-primed PCR revealed one patient with repeat expansion in *C9orf72.* Exome sequencing led to genetic diagnoses with variants in *MAPT* in 6 cases, *GRN* in 5 cases, *TBK1* in 5 cases, *OPTN* in 5 cases, *ANXA11* in 4 cases, *CHMP2B* in 3 cases, *SQSTM1* in 2 cases, *CYLD* in 2 cases, *TARDBP* in 2 cases, and *VCP*, *CCNF, SIGMAR1, CHCHD10, CHCHD2, FUS,* and *TMEM106B* in 1 case each. One case had a two octarepeats deletion in *PRNP,* and another case had a V180I mutation in *PRNP* (Fig. 2C). 29 variants have not been observed so far and can be considered novel, including the *MAPT* p.D54N, p.E342K, p.R221P, p.T263I, *TBK1* p.E696G, p.I37T, p.E232Q, p.S398F, p.T78A, p.Q150P, p.W259fs, *OPTN* p.R144G, p.F475V, *GRN* p.V473fs, p.C307fs, p.R101fs, *CHMP2B* p.K6N, p.R186Q, *ANXA11* p.Q155*, *CYLD* p.T157I, *SQSTM1* p.S403A, *UBQLN2* p.P509H, *CCNF* p.S160N, *CHCHD10* p.A8T, *SIGMAR1* p.S117L, *CHCHD2* p.P53fs, *FUS* p.S235G & p.S236G, and *TMEM106B* p.L144V variants. No dual diagnoses were made. Identified variants and their predicted pathogenicity are listed in Table 2. There was no statistical difference between the distribution of *ApoE* alleles in our FTD cohort and the control Chinese population (Table S2) [15].

**Table 2:** Interpretation of identified variants and their pathogenicity

**Gene**	**Mutation**	**Function**	**Novel/known**	**SNP-ID**	**Mutation Tasting/SIFT/Provean/PolyPhe*n*-2**	**CADD score**	**Clinvar**	**GnomAD, ExAC, 1000 Genomes Frequency**	**ACMG**	**Publication (PMID)**
*MAPT*	NM_005910 p.Asn279Lys/c.837 T > G	Missense	Known	Rs63750756	D/D/D/D	26.5	P	0,0,0	P: PS1 + PM1 + PM2 + PP3 + PP4	9,789,048
*MAPT*	NM_005910 p.Pro301Leu/c.902C > T	Missense	Known	Rs63751273	D/D/D/D	31	P	0,0,0	P: PS1 + PM1 + PM2 + PP3 + PP4	9,641,683
*MAPT*	NM_005910 p.Val337Met/c.1009G > A	Missense	Known	Rs63750570	D/D/D/D	26.9	P	0,0,0	P: PS1 + PM1 + PM2 + PP3 + PP4	9,629,852
*MAPT*	NM_005910 p.Asn296Asn/c.888T > C	Synonymous	Known	Rs63750912	N/T/N/B	0	P	0,0,0	P: PS1 + PM1 + PM2 + PP3 + PP4	11,117,553
*MAPT*	NM_005910 p.Gln351Arg/c.1052A > G	Missense	Known	NA	D/D/N/P	23.7	LP	0,0,0	P: PS1 + PM1 + PM2 + PP3 + PP4	23,998,300
*MAPT*	NM_005910 p.Arg5Cys/c.13C > T	Missense	Known	Rs766166210	D/D/D/P	27.8	VUS/B	3/250984, 2/118630,0	LP: PM1 + PM2 + PM5 + PP3 + PP4	25,604,855
*MAPT*	NM_005910 p.Arg5His/c.14G > A	Missense	Known	Rs63750959	D/T/N/B	22.9	LB/VUS/P	3/140076, 7/118676, 2/5008	P: PS1 + PS3 + PM1 + PM5 + PP4	11,921,059
*MAPT*	NM_005910 p.Asp54Asn/c.160G > A	Missense	Novel	NA	N/D/N/D	26.9	NA	0,0,0	LP: PM1 + PM2 + PP3 + PP4	NA
*MAPT*	NM_005910 p.Glu342Lys/c.1024G > A	Missense	Novel	NA	D/D/D/B	23.5	NA	0,0,0	LP: PM1 + PM2 + PM5 + PP3 + PP4	NA
*MAPT*	NM_005910 p.Ser285Arg/c.853A > C	Missense	Known	NA	D/D/D/D	27.8	NA	0,0,0	LP: PM1 + PM2 + PP1 + PP3 + PP4	25,443,551
*MAPT*	NM_005910 p.Arg221Pro/c.662G > C	Missense	Novel	NA	D/D/D/D	25.5	NA	0,0,0	LP: PM1 + PM2 + PM5 + PP3 + PP4	NA
*MAPT*	NM_005910 p.Gly389Arg/c.2341G > A	Missense	Known	Rs63750512	D/D/D/D	28.4	P	3/251208, 2/120720,0	P: PS1 + PS4 + PM1 + PM2 + PP3 + PP4	11,117,542
*MAPT*	NM_005910 p.Thr263Ile/c.788C > T	Missense	Novel	NA	D/D/D/D	24.2	NA	0,0,0	LP: PM1 + PM2 + PP3 + PP4	NA
*TBK1*	NM_013254 p.Glu696Gly/c.2087A > G	Missense	Novel	NA	D/T/D/P	29.3	NA	0,0,0	P: PS3 + PM2 + PM5 + PP3 + PP4	NA
*TBK1*	NM_013254 p.Ile37Thr/c.110T > C	Missense	Novel	NA	D/D/D/B	23.6	NA	0,0,0	P: PS3 + PM1 + PM2 + PM5 + PP3 + PP4	NA
*TBK1*	NM_013254 p.Glu232Gln/c.694G > C	Missense	Novel	NA	D/D/N/D	25.7	NA	0,0,0	P: PS3 + PM1 + PM2 + PP3 + PP4	NA
*TBK1*	NM_013254 p.Ser398Phe/c.1193C > T	Missense	Novel	NA	D/D/D/P	20.3	NA	0,0,0	LP: PS3 + PM2 + PP3 + PP4	NA
*TBK1*	NM_013254 p.Thr78Ala/c.232A > G	Missense	Novel	NA	D/T/D/B	22.5	NA	0,0,0	LP: PM1 + PM2 + PP3 + PP4	NA
*TBK1*	NM_013254 p.Gln150Pro/c.449A > C	Missense	Novel	NA	D/D/N/D	23.3	NA	0,0,0	LP: PM1 + PM2 + PP3 + PP4	NA
*TBK1*	NM_013254 p.Trp259GlyfsTer52/c.775del	Frameshift	Novel	NA	D/-/-/-	-	NA	0,0,0	P: PVS1 + PS3 + PM2 + PP3 + PP4	NA
*OPTN*	NM_021980 p.Leu494Trp/c.1481T > G	Missense	Known	Rs777195053	N/D/N/D	28.6	NA	1/140202, 1/121406, 0	P: PS1 + PM1 + PM2 + PP3 + PP4	24,908,169
*OPTN*	NM_021980 p.Glu516Gln/c.1546G > C	Missense	Known	Rs757107215	D/T/D/P	25.4	P	5/251460, 3/121358, 0	P: PS1 + PM1 + PM2 + PP3 + PP4	26,503,823
*OPTN*	NM_021980 p.Arg144Gly/c.430A > G	Missense	Novel	Rs1431906155	D/D/D/B	22.9	NA	1/251474/-/-	P: PS3 + PM1 + PM2 + PP3 + PP4	NA
*OPTN*	NM_021980 p.Phe475Val/c.1423T > G	Missense	Novel	NA	D/D/D/D	29.5	NA	0,0,0	P: PS3 + PM1 + PM2 + PP3 + PP4	NA
*OPTN*	NM_021980 p.Thr282Pro/c.844A > C	Missense	Known	Rs773125318	N/T/D/B	7.139	NA	8/251478,5/121294,0	LP: PM1 + PM2 + PP4 + PP5	21,613,650
*OPTN*	NM_021980 p. Ala136Val/c.407C > T	Missense	Known	Rs764364218	N/T/D/B	8.1	VUS	16/251442,4/121362,0	LP: PM1 + PM2 + PP4 + PP5	26,503,823
*GRN*	NM_002087 p.Pro451Leu /c.1352C > T	Missense	Known	Rs752428000	D/D/N/D	28.1	VUS	1/140268, 1/120150, 0	LP: PM1 + PM2 + PP3 + PP4 + PP5	18,565,828
*GRN*	NM_002087 p.Val473fs/c.1414-14_1444del	Deletion/Frameshift	Novel	NA	D/-/-/-	-	NA	0,0,0	P: PVS1 + PS3 + PM2 + PP3 + PP4	NA
*GRN*	NM_002087 p.Thr487Ile/c.1460C > T	Missense	Known	Rs772784579	D/D/D/P	25.4	VUS	2/140306,0,0	LP: PM1 + PM2 + PP3 + PP4 + PP5	29,530,724
*GRN*	NM_002087 p.Cys307GlufsTer3/c.914_915ins	Insertion/Frameshift	Novel	NA	D/-/-/-	-	NA	0,0,0	P: PVS1 + PS3 + PM2 + PP3 + PP4	NA
*GRN*	NM_002087 p.Asn119del/c.355_357del	Deletion	Known	Rs758168578	D/-/-/-	-	VUS	6/140088, 8/121346, 1/6404	LP: PS4 + PM4 + PP4 + PP5	29,339,765
*GRN*	NM_002087 p.Arg101GlnfsTer13/c.302_315del	Deletion/Frameshift	Novel	NA	D/-/-/-	-	NA	0,0,0	P: PVS1 + PS3 + PM2 + PP3 + PP4	NA
*ANXA11*	NM_001157 p.Asp40Gly/c.119A > G	Missense	Known	Rs1247392012	D/T/D/P	23.1	P	2/174858,0,0	LP: PM1 + PM2 + PP2 + PP3 + PP4 + PP5	33,087,501
*ANXA11*	NM_001157 p.Gln155*/c.463C > T	Nonsense	Novel	NA	D/-/-/-	-	NA	0,0,0	P: PVS1 + PM2 + PP3 + PP4	NA
*ANXA11*	NM_001157 p.Pro36Arg/c.107C > G	Missense	Known	Rs199988035	D/D/D/D	23.5	NA	0,0,0	LP: PM1 + PM2 + PP3 + PP4 + PP5	36,458,208
*CHMP2B*	NM_014043 p.Arg186Gln/c.557G > A	Missense	Novel	Rs747423794	D/T/D/B	21.3	NA	0, 2/119998, 0	LP: PM1 + PM2 + PP3 + PP4	NA
*CHMP2B*	NM_014043 p.Arg205Trp/c.613C > T	Missense	Known	Rs373536428	D/D/D/B	23.1	VUS	12/250486,9/120064,0	LP: PM1 + PM2 + PP3 + PP4 + PP5	29,411,640
*CHMP2B*	NM_014043 p.Lys6Asn/c.18G > T	Missense	Novel	NA	D/D/D/D	23.3	NA	0,0,0	LP: PM1 + PM2 + PP3 + PP4	NA
*CYLD*	NM_015247 p.Gln443Lys/c.1327C > A	Missense	Known	Rs764952788	D/T/D/P	22.2	NA	4/249318,3/120728,0	LP: PM1 + PM2 + PP3 + PP4 + PP5	34,868,212
*CYLD*	NM_015247 p.Thr157Ile/c.470C > T	Missense	Novel	NA	D/D/D/P	21.9	NA	0,0,0	LP: PM1 + PM2 + PP3 + PP4	NA
*SQSTM1*	NM_003900 p.Glu362Lys/c.1084G > A	Missense	Known	Rs535932454	D/D/N/P	23	VUS	1/140202, 4/121302, 1/5008	LP: PS4 + PM1 + PP3 + PP4	31,859,009
*SQSTM1*	NM_003900 p.Ser403Ala/c.1207T > G	Missense	Novel	NA	D/T/N/D	26.3	VUS	0,0,0	LP: PM1 + PM2 + PP3 + PP4	NA
*PRNP*	NM_000311 p.Val180Ile/c.538G > A	Missense	Known	Rs74315408	D/D/D/D	22.5	P/LP	4/140106, 6/121398, 1/5008	P: PS1 + PS3 + PM1 + PM2 + PP3 + PP4 + PP5	20,301,407
*PRNP*	NM_000311 2-OPRD	Deletion	Known	Rs193922906	-	-	VUS	0,0,0	LP: PM1 + PM2 + PP4 + PP5	12,451,210; 11,468,331
*VCP*	NM_007126 p.Arg662Cys/c.1984C > T	Missense	Known	Rs765795425	D/D/D/D	31	VUS	1/249512,1/120766,0	LP: PM1 + PM2 + PP3 + PP4 + PP5	22,572,540
*TARDBP*	NM_007375 p.Ile383Val/c.1147A > G	Missense	Known	Rs80356740	D/T/N/B	17.19	P	4/230726, 1/115618, 0	LP: PS1 + PM1 + PP2 + PP4	18,802,454; 26,581,115; 30,773,994
*UBQLN2*	NM_013444 p.Pro509His/c.1526C > A	Missense	Novel	Rs868418213	N/D/N/P	23.4	NA	0,0,0	LP: PM2 + PM5 + PP2 + PP3 + PP4	NA
*CCNF*	NM_001761 p.Ser160Asn/c.479G > A	Missense	Novel	NA	D/T/D/D	19.6	NA	0,0,0	LP: PM1 + PM2 + PP3 + PP4	NA
*CHCHD10*	NM_213720 p.Ala8Thr/c.22G > A	Missense	Novel	NA	N/T/D/B	11.57	NA	0,0,0	LP: PM1 + PM2 + PP3 + PP4	NA
*SIGMAR1*	NM_005866 p.Ser117Leu/c.350C > T	Missense	Novel	NA	D/D/D/D	33	NA	0,0,0	LP: PM1 + PM2 + PP3 + PP4	NA
*CHCHD2*	NM_001320327 p.Pro53fs/c.153_156dupGCAG	Frameshift	Novel	NA	D/-/-/-	-	NA	0,0,0	P: PVS1 + PM2 + PP3 + PP4	NA
*FUS*	NM_004960 p.Ser235Gly/c.703A > G NM_004960 p.Ser236Gly/c.706A > G	Missense Missense	Novel Novel	NA NA	N/T/D/B N/T/D/B	17.33 17.23	NA NA	0,0,0 0,0,0	LP: PM1 + PM2 + PP3 + PP4 LP: PM1 + PM2 + PP3 + PP4	NA NA
*TMEM106B*	NM_001134232 p.Leu144Val/c.430T > G	Missense	Novel	NA	D/D/D/P	24.7	NA	0,0,0	LP: PM1 + PM2 + PP2 + PP3 + PP4	NA

Mutationtaster: *D* Disease causing,* N*  Polymorphism, *SIFT: D*  Damaging,* T* Tolerated, Provean: *D* Deleterious, *N* Neutral, Polyphen-2: *D* Probably damaging, *P*  Possibly damaging, *B* Benign, Clinvar/ACMG: P  Pathogenic, *LP*  Likely pathogenic, *VUS* Variants of uncertain significance, *LB* Likely benign

### Clinical findings of *TBK1* and *OPTN* variants

**Fig. 3:**
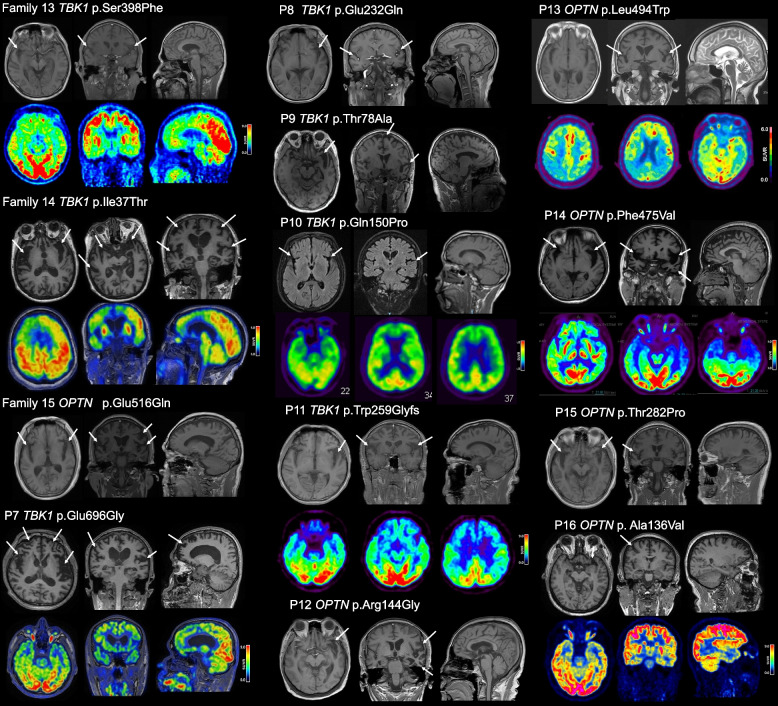
Neuroimaging studies of the FTD patients with *TBK1* and *OPTN* variants. The upper lane shows the cranial MRI. The atrophic changes in frontotemporal regions are indicated by white arrows. The lower lane shows the ^18^F-FDG-PET images. The color bar of the ^18^F-FDG-PET images indicates the corresponding regional standardized uptake value ratios (SUVR)

The clinical characteristics of 61 FTD-gene variant carriers are summarized in Table 3. In this study, we focus on the clinical characteristics of the patients with *TBK1* and *OPTN* variants. Figure 3 presents the neuroimaging results of cranial MRI or ^18^F-FDG-PET of the patients with *TBK1* and *OPTN* variants.

**Table 3:** The correlation between genetic features and clinical manifestations of 28 FTD-gene variant carriers

ID	*Gene*	Mutation		Sex	Age	AAO	Duration	Clinical phenotype	MMSE	MoCA	Cranial MRI	^18^F-FDG-PET
Family 1	*C9orf72*		Hexanucleotide repeat expansion	Female	68	65	3	FTD-P	13	5	bilateral frontal and temporal lobe atrophy	NA
Family 2	*MAPT*		p.Pro301Leu/c.902C > T	Female	56	53	3	bvFTD	16	11	bilateral frontal and temporal lobe atrophy	NA
Family 3	*MAPT*		p.Asn279Lys/c.837 T > G	Female	48	46	2	FTD-P	15	6	bilateral frontal and temporal lobe atrophy	NA
Family 4	*MAPT*		p.Pro301Leu/c.902C > T	Male	64	63	1	bvFTD	14	6	bilateral frontal and temporal lobe atrophy	NA
Family 5	*MAPT*		p.Val337Met/c.1009G > A	Female	56	52	4	bvFTD	10	8	Bilateral temporal lobe atrophy	NA
Family 6	*MAPT*		p.Asn296Asn/c.888T > C	Male	47	46	1	bvFTD	27	19	bilateral frontal and temporal lobe atrophy	NA
Family 7	*MAPT*		p.Pro301Leu/c.902C > T	Female	57	52	5	bvFTD	12	5	bilateral frontal and temporal lobe atrophy	NA
Family 8	*MAPT*		p.Arg5Cys/c.13C > T	Male	44	44	0.5	bvFTD	20	16	mild atrophy of the bilateral frontal and temporal lobes	hypoperfusion in bilateral frontal and temporal lobes
Family 9	*MAPT*		p.Gly389Arg/c.2341G > A	Male	39	37	2	bvFTD	8	3	bilateral frontal and temporal lobe atrophy	NA
Family 10	*MAPT*		p.Glu342Lys/c.1024G > A	Female	59	57	2	FTD-P	17	9	bilateral frontal and temporal lobe atrophy	NA
Family 11	*MAPT*		p.Gln351Arg/1052A > G	Female	54	51	3	bvFTD	11	5	bilateral frontal and temporal lobe atrophy	NA
Family 12	*MAPT*		p.Ser285Arg/c.853A > C	Male	39	35	4	FTD-P	15	6	midbrain atrophy	NA
Family 13	*TBK1*		p.Ser398Phe/c.1193C > T	Male	63	54	9	svPPA	19	10	bilateral frontal and temporal lobe atrophy	hypoperfusion in bilateral frontal and temporal lobes
Family 14	*TBK1*		p.Ile37Thr/c.110T > C	Male	55	50	5	svPPA	15	9	bilateral frontal, temporal, and parietal lobe atrophy	hypoperfusion in bilateral frontal and temporal lobes, more severe on the right
Family 15	*OPTN*		p.Glu516Gln/c.1546G > C	Male	67	64	3	FTD-P	10	4	bilateral frontal and temporal lobe atrophy	NA
Family 16	*GRN*		p.Thr487Ile/c.1460C > T	Male	56	52	4	bvFTD	14	6	bilateral frontal and temporal lobe atrophy	NA
Family 17	*UBQLN2*		p.Pro509His/c.1526C > A	Male	61	53	8	bvFTD	12	5	bilateral frontal and temporal lobe atrophy	NA
P1	*MAPT*		p.Asp54Asn/c.160G > A	Female	70	67	3	bvFTD	14	4	bilateral frontal and temporal lobe atrophy	hypoperfusion in bilateral frontal and temporal lobes
P2	*MAPT*		p.Gln351Arg/c.1052A > G	Female	47	45	2	bvFTD	26	25	bilateral frontal and temporal lobe atrophy	NA
P3	*MAPT*		p.Arg5His/c.14G > A	Female	52	49	3	bvFTD	9	1	bilateral frontal and temporal lobe atrophy	NA
P4	*MAPT*		p.Arg5His/c.14G > A	Female	67	61	6	bvFTD	13	4	bilateral frontal and temporal lobe atrophy	NA
P5	*MAPT*		p.Arg221Pro/c.662G > C	Female	74	70	4	bvFTD	12	5	bilateral frontal and temporal lobe atrophy	NA
P6	*MAPT*		p.Thr263Ile/c.788C > T	Male	61	4	57	bvFTD	22	17	bilateral frontal and temporal lobe atrophy	NA
P7	*TBK1*		p.Glu696Gly/c.2087A > G	Female	69	65	5	bvFTD	11	4	atrophy in the bilateral frontal and temporal lobes	hypoperfusion in bilateral frontal and temporal lobes
P8	*TBK1*		p.Glu232Gln/c.694G > C	Female	63	56	7	FTD-P	25	22	mild and aspecific cortical atrophy	NA
P9	*TBK1*		p.Thr78Ala/c.232A > G	Female	70	67	3	svPPA	19	14	left frontal and temporal lobe atrophy	NA
P10	*TBK1*		p.Gln150Pro/c.449A > C	Female	64	62	2	bvFTD	19	12	bilateral frontal and temporal lobe atrophy	hypoperfusion in bilateral frontal and temporal lobes
P11	*TBK1*		p.Trp259GlyfsTer52/c.775del	Male	50	49	1	FTD-ALS	18	8	bilateral frontal, temporal, and parietal lobe atrophy	hypoperfusion in the bilateral frontal, parietal, and temporal lobes
P12	*OPTN*		p.Arg144Gly/c.430A > G	Female	60	58	2	svPPA	9	8	bilateral temporal lobe atrophy, more severe on the left	NA
P13	*OPTN*		p.Leu494Trp/c.1481T > G	Female	63	61	2	svPPA	13	5	mild cortical atrophy in the bilateral temporal lobes	hypoperfusion in bilateral temporal and parietal lobes
P14	*OPTN*		p.Phe475Val/c.1423T > G	Female	48	47	1	svPPA	17	10	asymmetric frontal and temporal lobe atrophy, more severe on the left	hypoperfusion in bilateral frontal and temporal lobes
P15	*OPTN*		p.Thr282Pro/c.844A > C	Male	54	51	3	svPPA	25	19	bilateral frontal and temporal lobe atrophy, more severe on the right	
P16	*OPTN*		p. Ala136Val/c.407C > T	Female	53	51	2	bvFTD	14	7	bilateral frontal and temporal lobe atrophy	mild hypoperfusion in bilateral temporal lobes
P17	*GRN*		p.Pro451Leu/c.1352C > T	Male	52	51	1	bvFTD	23	19	mild bilateral frontal and temporal lobe atrophy	hypoperfusion in bilateral frontal and temporal lobes
P18	*GRN*		p.Val473fs/c.1414-14_1444del	Female	57	57	1	bvFTD	11	6	bilateral frontal and temporal lobe atrophy	NA
P19	*GRN*		p.Cys307GlufsTer3/c.914_915ins	Male	58	54	4	bvFTD	15	10	bilateral frontal and temporal lobe atrophy	NA
P20	*GRN*		p.Asn119del/c.355_357del	Male	65	63	2	bvFTD	23	17	bilateral frontal and temporal lobe atrophy	NA
P21	*GRN*		p.Arg101GlnfsTer13/c.302_315del	Female	64	61	3	bvFTD	9	4	bilateral frontal and temporal lobe atrophy	NA
P22	*ANXA11*		p.Asp40Gly/c.119A > G	Female	51	49	2	bvFTD	19	12	bilateral frontal and temporal lobe atrophy	NA
P23	*ANXA11*		p.Gln155*/c.463C > T	Male	56	52	4	svPPA	23	18	bilateral temporal lobe atrophy	NA
P24	*ANXA11*		p.Pro36Arg/c.107C > G	Male	73	69	4	bvFTD	21	15	left temporal lobe atrophy	NA
P25	*ANXA11*		p.Pro36Arg/c.107C > G	Female	75	69	6	FTD-ALS	18	13	bilateral frontal lobe atrophy	NA
P26	*PRNP*		p.Val180Ile/c.538G > A	Male	66	64	2	bvFTD	15	7	bilateral frontal and temporal lobe atrophy	NA
P27	*PRNP*		2-OPRD R2 R3	Female	74	70	4	bvFTD	25	20	bilateral frontal and temporal lobe atrophy	NA
P28	*CHMP2B*		p.Arg186Gln/c.557G > A	Male	59	57	2	bvFTD	15	9	bilateral temporal lobe atrophy	NA
P29	*CHMP2B*		p.Arg205Trp/c.613C > T	Female	65	63	2	bvFTD	22	19	bilateral frontal and temporal lobe atrophy	NA
P30	*CHMP2B*		p.Lys6Asn/c.18G > T	Female	42	39	3	bvFTD	15	9	bilateral frontal and temporal lobe atrophy	NA
P31	*SQSTM1*		p.Ser403Ala/c.1207T > G	Female	56	55	1	FTD-ALS	8	4	bilateral frontal lobe atrophy	NA
P32	*SQSTM1*		p.Glu362Lys/c.1084G > A	Female	53	51	2	bvFTD	13	5	mild bilateral frontal and temporal lobe atrophy	mild hypoperfusion in bilateral frontal and temporal lobes, more severe on the right
P33	*CYLD*		p.Gln443Lys/c.1327C > A	Male	69	65	4	bvFTD	21	17	bilateral frontal and temporal lobe atrophy	NA
P34	*CYLD*		p.Thr157Ile/c.470C > T	Male	48	46	2	bvFTD	11	4	bilateral frontal and temporal lobe atrophy	NA
P35	*C9orf72*		Hexanucleotide repeat expansion	Male	47	46	1	bvFTD	14	6	bilateral frontal and temporal lobe atrophy	hypoperfusion in bilateral frontal and temporal lobes
P36	*TARDBP*		p.Ile383Val/c.1147A > G	Male	74	70	4	bvFTD	20	16	bilateral frontal and temporal lobe atrophy	NA
P37	*TARDBP*		p.Ile383Val/c.1147A > G	Male	67	62	5	FTD-ALS	23	18	bilateral frontal lobe atrophy	NA
P38	*VCP*		p.Arg662Cys/c.1984C > T	Male	73	70	3	svPPA	16	15	bilateral frontal and temporal lobe atrophy, more severe on the left	NA
P39	*CCNF*		p.Ser160Asn/c.479G > A	Female	69	67	2	bvFTD	3	2	left frontal and temporal lobe atrophy	NA
P40	*SIGMAR1*		p.Ser117Leu/c.350C > T	Male	64	61	3	bvFTD	19	15	bilateral frontal and temporal lobe atrophy	NA
P41	*CHCHD10*		p.Ala8Thr/c.22G > A	Male	66	63	3	bvFTD	4	2	bilateral frontal and occipital lobe atrophy	NA
P42	*CHCHD2*		p.Pro53fs/c.153_156dupGCAG	Female	67	64	3	bvFTD	22	18	bilateral frontal and temporal lobe atrophy	NA
P43	*FUS*		p.Ser235Gly/c.703A > G p.Ser236Gly/c.706A > G	Male	70	67	3	bvFTD	10	4	left temporal lobe atrophy	NA
P44	*TMEM106B*		p.Leu144Val/c.430T > G	Male	62	59	3	bvFTD	11	5	bilateral frontal and temporal lobe atrophy	NA

*bvFTD* behavioral variant of FTD, *svPPA* semantic variant of primary progressive aphasia, *AAO* age at onset, *APOE* *apolipoprotein E*, *MMSE* Mini-Mental State Examination, *MoCA* Montreal Cognitive Assessment Test, *OPRD* octapeptide repeat deletion, *MRI* magnetic resonance imaging examinations, ^*18*^*F-FDG PET* ^18^F-fluorodeoxyglucose positron emission tomography, *NA* assessment could not be done because the patient was uncooperative or neuroimaging data was unavailable

#### TBK1 variants

##### Family 13 *TBK1* p.Ser398Phe/c.1193C > T

The proband, a 63-year-old male, began experiencing memory loss, a change in personality, frequent mood swings, and difficulty expressing and understanding speech at the age of 54. Over time, he gradually developed an inability to recognize family members and perform activities of daily living. He was diagnosed with svPPA [2]. The patient's father also exhibited similar symptoms and was diagnosed with "dementia" at the age of 70. He subsequently passed away due to an accident at the age of 73.

##### Family 14 *TBK1* p.Ile37Thr/c.110 T > C

The proband of this family was a 55-year-old male with a five-year history of anomia and personality changes. He presented with progressive fluent aphasia with impaired naming and loss of understanding of even single words five years ago. On examination, there were also extrapyramidal symptoms of rigidity. The diagnosis of svPPA was confirmed [2]. The patient’s father and older sister all presented similar symptoms.

##### P7 *TBK1* p.Glu696Gly/c.2087A > G

The patient exhibited memory problems and personality changes, with initial short-term memory loss leading to difficulty retaining new information. Subsequently, she struggled with daily practical tasks and experienced behavioral disturbances such as stubbornness and repetitive behaviors. She also showed a decline in personal hygiene and reluctance to engage in self-care. She was diagnosed with bv-FTD [18].

##### P8 *TBK1* p.Glu232Gln/c.694G > C

The patient presented language abnormalities manifesting as deficits in speech production and was initially diagnosed with nvPPA [2]. The patient then exhibited extrapyramidal symptoms of rigidity and bradykinesia in the right limbs, but no personality changes or behavioral abnormalities were noted. The diagnosis was subsequently revised to FTD-P [20].

#####  P9 *TBK1* p.Thr78Ala/c.232A > G

The patient presented with a decline in verbal communication accompanied by a decrease in memory function at the age of 67. This decline includes forgetting the names of individuals and places, as well as an inability to recognize written language. She was diagnosed with svPPA [2].

##### P10 *TBK1* p.Gln150Pro/c.449A > C

The patient presented with anxiety accompanied by reduced verbal communication and memory decline. The onset of anxiety occurred at the age of 63, characterized by worries and fears, primarily concerning the possibility of having a serious illness and fear of impending death. Family members gradually noticed changes in the patient's personality and reduced speech output. Additionally, there was evident memory decline, and a notable slowing of response and movement, accompanied by a decline in comprehension and computational abilities. She was diagnosed with bv-FTD [18].

##### P11 *TBK1* p.Trp259GlyfsTer52/c.775del

A male patient, aged 50, has been experiencing unclear speech accompanied by decreased memory for over six months. Six months ago, the patient exhibited unclear speech characterized by indistinct articulation and a hoarse voice. The patient experiences coughing when drinking water and also reports difficulty swallowing. Grip strength in both hands has diminished, evidenced by an inability to operate a lighter and difficulty opening packaged items, while lower limb strength remains unchanged. Memory decline is notable, primarily affecting recent events. The patient has become more introverted, displaying a reduced inclination for communication and social interaction. He was diagnosed with FTD-ALS [19].

#### OPTN variants

##### Family 15 *OPTN* p.Glu516Gln/c.1546G > C

The proband exhibited progressive speech and language deficits, with difficulty expressing thoughts and finding words, as well as errors in speech sounds and sentence construction. Additionally, he developed PSP-like symptoms, including mildly elevated axial muscle tone in the trunk and hips, and vertical gaze palsy. He was clinically diagnosed with FTD-P [20]. His older brother was reported to have similar symptoms, but could not be examined. The medical history of their parents, both of whom died at an early age, was unavailable.

##### P12 *OPTN* p.Arg144Gly/c.430A > G

The patient exhibited impaired memory and language difficulties, manifesting as short-term memory impairment, difficulty naming objects, comprehending word meanings, and understanding spoken or written language. She was clinically diagnosed with svPPA [2].

##### P13 *OPTN* p.Leu494Trp/c.1481 T > G

The patient initially presented with forgetfulness, language difficulties, and personality changes, including short-term memory impairment, difficulty naming familiar items, and struggling to find the right words. With disease progression, she exhibited obsessive behaviors, fear of leaving home, and disinhibition. Her speech became increasingly vague and she experienced difficulty carrying out daily activities. She was clinically diagnosed with svPPA [2].

#####  P14 *OPTN* p.Phe475Val/c.1423 T > G

The patient first developed a memory deficit and depression, with rapid progression of behavioral symptoms, including irritability, anxiety, and apathy. She then exhibited impaired word-finding, deteriorating naming abilities, and progressive difficulty comprehending spoken or written language, with unreasonable responses. Disorientation in time and space also manifested. She was clinically diagnosed with svPPA [2].

##### P15 *OPTN* p.Thr282Pro/c.844A > C

The patient, currently 54 years old, progressively displayed changes in personality and behavior, including stubbornness, irritability, diminished concern for family members, disregard for others' feelings, and a lack of social etiquette since the age of 51. Furthermore, symptoms comprised declining recent memory, reduced executive function, impaired language abilities, and challenges with naming and writing. He was clinically diagnosed with svPPA [2].

##### P16 *OPTN* p. Ala136Val/c.407C > T

The patient experienced memory decline at the age of 51, predominantly affecting recent memory, characterized by an inability to recall recent events and difficulty remembering familiar names. The patient also exhibited personality changes. She experienced behavioral disturbances such as stubbornness and repetitive behaviors. She was diagnosed with bv-FTD [18].

### Functional analysis of TBK1 variants

**Fig. 4:**
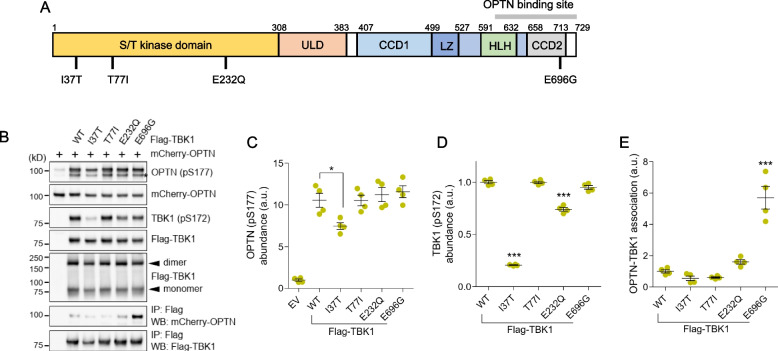
Biochemical characterization of TBK1 variants. **A** Domain structure and OPTN-binding site of TBK1 protein. The variants identified in this study are indicated. **B** Top five lanes: HEK293T cells expressing Flag-TBK1 wild-type and indicated mutants were co-transfected with mCherry-OPTN for 48 h before lysis. The binding of TBK1 variants was analyzed via immunoblot (IB). The asterisk points to OPTN pS177 bands. Bottom two lanes: Coimmunoprecipitation (co-IP) of Flag-TBK1 (wild-type and indicated mutants) and mCherry-OPTN wild-type from HEK293T cell lysates. **C** Phosphorylation of OPTN S177 was confirmed by IB using the pS177 OPTN antibody generated by immunoGlobe GmbH. EV: empty vector. **D** Cell lysates were subjected to immunoblot analysis and active TBK1 (pS172) was detected with a phosphospecific antibody (pS172; no. 5483; Cell Signaling Technology). **E** Co-immunoprecipitation (Co-IP) of Flag-TBK1 wild-type and the indicated mutants with mCherry-OPTN wild-type from HEK293T cells using an antibody to Flag. Co-immunoprecipitated proteins were analyzed by immunoblotting (IB) with the Flag and mCherry antibodies

Serine/threonine-protein kinase TBK1 regulates selective autophagy pathways, specifically mitophagy [21] and xenophagy [22]. TBK1 phosphorylates a selective autophagy receptor, OPTN, at Ser177, that enhances interaction with ubiquitinated cargoes and is an autophagy modifier of the LC3 family [23]. The three mutations of TBK1 p.I37T, p.T77I, and p.E232Q were located in the kinase catalytic domain, and p.E696G was located in the coiled-coil 2 (CCD2) domain involved in binding with OPTN (Fig. 4A). We examined whether TBK1 and its mutants actively phosphorylated and associated with OPTN in the HEK293T cells transfected with TBK1 and OPTN expression plasmids. The T77I variant found in a normal elderly person without symptoms was used as a control. Overexpression of wild-type TBK1 robustly phosphorylated OPTN. However, OPTN phosphorylation by the TBK1 I37T mutant was significantly reduced compared to the wild type (Fig. 4B, C). Generally, autophosphorylation of kinases is defined as the phosphorylation of kinase itself, which normally regulates the catalytic activity. We next examined the autophosphorylation (pS172) of wild-type and mutants TBK1. Autophosphorylation of the mutant I37T was significantly reduced (Fig. 4D). This was consistent with the result of the OPTN-phosphorylation. Autophosphorylation of the E232Q was also reduced (Fig. 4D), but it did not affect the OPTN-phosphorylation. It is known that dimerization of TBK1 is required for kinase activation and autophosphorylation [24]. Therefore, we next examined TBK1-dimerization using non-reducing SDS-PAGE. Although the autophosphorylation and kinase activity for OPTN were reduced, the dimerization ability of the I37T mutant and the other mutants were not changed compared to wild-type TBK1 (Fig. 4B).

The mutation E696G is located in the CCD2 domain, which involves interaction with OPTN (Fig. 4A) [25]. We next examined TBK1-OPTN interaction using immunoprecipitation. It was found that OPTN-TBK1 complex formation is significantly enhanced in cells transfected with human TBK1 E696G mutant (Fig. 4B, E). The TBK1 E232Q mutant also slightly increased the OPTN-TBK1 complex formation, and the TBK1 I37T mutant marginally decreased the OPTN-TBK1 binding, but these effects did not reach statistical significance (Fig. 4B, E).

### Functional analysis of OPTN variants

**Fig. 5:**
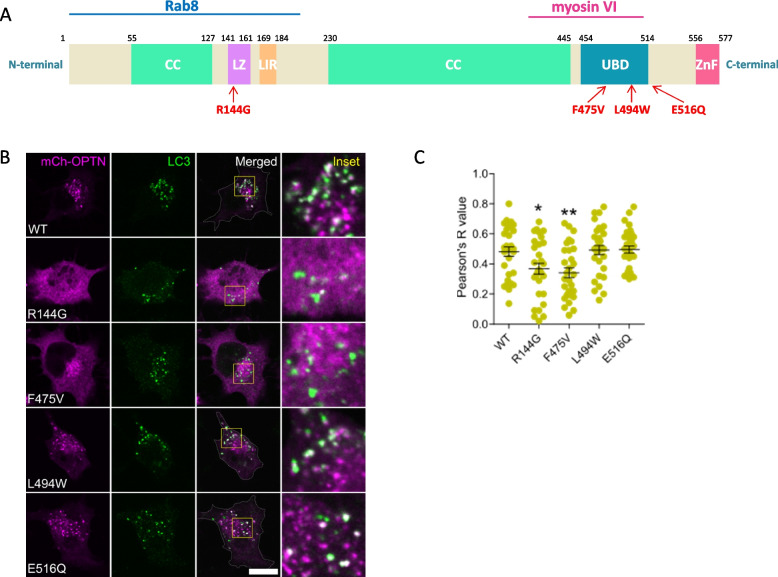
Recruitment of OPTN variants to the autophagosomes. **A** Schematic representation of domain structure in human optineurin protein and the localization of these domains relative to its amino acid sequence. CC, coiled-coil; LZ, leucine zipper domain; LIR, LC3-interacting region; UBD, ubiquitin-binding domain; ZnF, zinc finger. Rab8-interacting region (amino acids 1–209) and myosin VI-interacting region (amino acids 417–512) were indicated. The mutations identified in our study are indicated by red arrows.** B** Representative confocal images in Neuro2a cells showing co-localization of endogenous LC3 puncta (green) with overexpressed OPTN (magenta) wild-type and mutants. In Neuro2A cells transfected with OPTN wild-type, L494W, and E516Q mutants, optineurin-positive vesicles co-localized with LC3. In Neuro2A cells expressing the OPTN R144G and F475V mutants, optineurin is not vesicular and displayed decreased colocalization with LC3. Insets show higher magnification of the areas outlined in the merged images. Scale bar: 10 μm. **C** The co-localization of OPTN wild-type or mutants with LC3-positive autophagosome. WT = wild-type. Pearson's *R* values between LC3 puncta (autophagosomes) and mCherry-OPTN proteins were shown. *n* = 30 cells. * *p* < 0.05, ** *p* < 0.01

OPTN is an autophagy adaptor protein that plays a critical role in multiple stages of the autophagic pathway. In addition, it is associated with several human disorders that are closely linked to autophagy. It contains two coiled-coil domains, a leucine zipper (LZ) domain, a microtubule-associated protein 1 light chain (LC3)-interacting region (LIR), a ubiquitin-binding domain (UBD), and a zinc finger (ZnF) domain [26] (Fig. 5A). Previous studies have demonstrated that ALS-linked OPTN mutants (E478G and Q398X) prevented vesicle formation and induced non-vesicular localization of optineurin in cells [27]. We investigated whether OPTN mutants identified in our FTD cohort could be recruited to autophagosomes in Neuro2a cells. Exogenous expression of OPTN wild-type, L494W, and E516Q resulted in the formation of vesicular structures that colocalized with LC3 puncta. In contrast, OPTN R144G and F475V mutants were significantly diffused throughout the cytoplasm ((Fig. 5B, C). Our image analysis revealed a significant reduction in the recruitment to LC3 puncta compared to OPTN wild-type (Fig. 5B, C) indicating functional defects as autophagy adaptor proteins.

### The frequencies of genes implicated in FTD in China

**Fig. 6:**
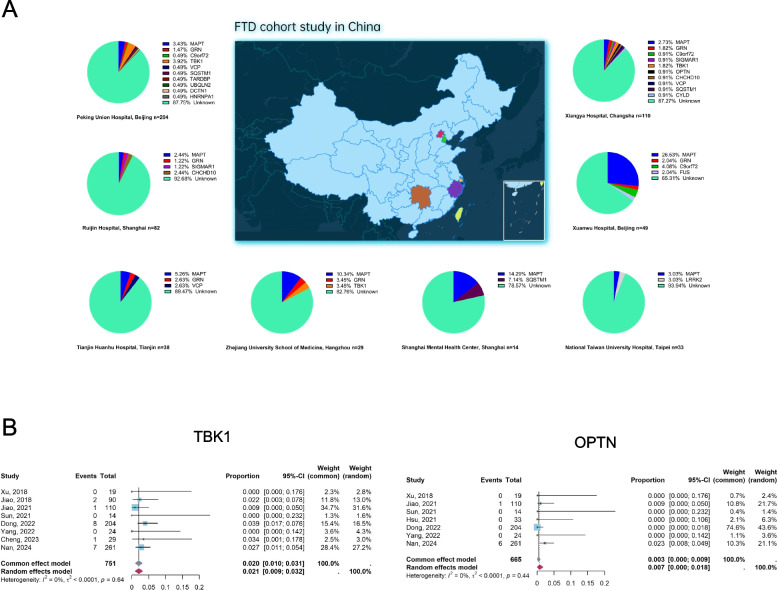
The genetic spectrum and the variant frequency of Chinese FTD patients. **A **The genetic spectrum of the major FTD cohorts in China. **B** The overall variant frequency of *TBK1* and *OPTN* in Chinese FTD patients

In the literature review, rare variants in genes such as *MAPT, GRN, C9orf72, CHCHD10, VCP, TBK1, OPTN, SQSTM1, SIGMAR1, TARDBP, UBQLN2, FUS, CCNF,* and *CYLD* were identified in Chinese FTD populations [8, 28–36]. The genetic spectrum of the major FTD cohorts previously reported in China is shown in (Fig. 6A). Including the patients identified in our cohort, the top six genes with the highest frequency implicated in FTD in China are shown in Table 4. The pooled frequency of *TBK1* and *OPTN* in Chinese FTD patients was 2.0% (CI: 1.0%-3.1%) and 0.3% (CI: 0.0%-0.9%), respectively (Fig. 6B). No substantial heterogeneity was found in the pooled frequency meta-analysis for both *TBK1* and *OPTN* across all datasets (*I*^*2*^: 0%). Notably, the frequency of *TBK1* mutations was the second highest among Chinese FTD patients, surpassing the mutation frequencies of *GRN* (1.7%) and *C9orf72* (0.5%).

**Table 4:** The top six genes with the highest frequency implicated in FTD in China

Genes	Studies	Positive cases	Total Cases	Mutation rate (95%CI)	Q-value	*p* (Q)	*I*^*2*^ (%)	*p*_*f*_ value
*MAPT*	10	40	842	0.037 (0.025 to 0.050)	9.57	0.3868	5.90%	0.094
*TBK1*	8	19	751	0.020 (0.010 to 0.031)	5.14	0.6426	0.00%	0.7709
*GRN*	11	16	866	0.017 (0.008 to 0.026)	2.73	0.9871	0.00%	0.9349
*C9orf72*	11	6	846	0.005 (0.000 to 0.011)	2.73	0.987	0.00%	0.5501
*OPTN*	7	7	665	0.003 (0.000 to 0.009)	5.88	0.4362	0.00%	0.5131
*CHCHD10*	8	9	779	0.003 (0.000 to 0.008)	7.86	0.3451	10.90%	0.1355

*FTD* frontotemporal dementia, *Q* heterogeneity between studies, *p (Q)* *p*-value for heterogeneity, *I*^*2*^ percentage of heterogeneity caused by study differences, *p*_*f*_: *p*-value for linear regression test of funnel plot asymmetry

## Discussion

This study represents the largest FTD cohort in China and the first cohort study to conduct comprehensive screening for causative mutations, investigating mutation frequencies and underlying pathogenesis in Chinese FTD patients. We conducted functional validation of the pathogenic variants in *TBK1* and *OPTN*, indicating that *TBK1* and *OPTN* variants could potentially be a common cause of FTD in the Chinese population.

In this study, FTD-gene mutations are detected in 23.4% (61/261) of FTD patients, which is higher than the previous findings [8]. 90.8% (237/261) of dementia patients in our cohort have an early-onset age, which may partially explain the relatively high rate of genetic diagnosis. In addition to exome sequencing which is routinely performed, we also performed nested PCR, agarose electrophoresis, and repeat-primed PCR to detect the presence of the repeat expansions in the *PRNP* and *C9orf72* genes*.* Consistent with prior genetic studies on Chinese FTD patients [8, 35], our analysis revealed rare variants in *MAPT*, *GRN*, *TBK1*, *PRNP,* and the GGGGCC repeats in the *C9orf72*. Additionally, we observed rare variants in *OPTN*, *ANXA11, CHMP2B, CYLD, SQSTM1, VCP, TARDBP*, *UBQLN2*, *CCNF*, *CHCHD10, SIGMAR1, CHCHD2, FUS,* and *TMEM106B*.

In total, 29 novel pathogenic or likely pathogenic variants are found in our cohort. The *GRN* V473fs, p.C307fs, p.R101fs, *TBK1* p.W259fs, *ANXA11* p.Q155*, and *CHCHD2* p.P53fs variants are either frameshift or nonsense variants that may lead to truncated proteins or affect splicing. The *MAPT* p.D54N, p.E342K, p.R221P, p.T263I, *TBK1* p.E696G, p.I37T, p.E232Q, p.S398F, p.T78A, p.Q150P, *OPTN* p.R144G, p.F475V, *CHMP2B* p.R186Q, p.K6N, *CYLD* p.T157I, *SQSTM1* p.S403A, *UBQLN2* p.P509H, *CCNF* p.S160N, *CHCHD10* p.A8T, *SIGMAR1* p.S117L, *FUS* p.S235G & p.S236G, and *TMEM106B* p.L144V variants are rare missense variants found in a highly conserved region, absent from gnomAD or controls, and predicted to be deleterious by in silico algorithms. According to the ACMG criteria [14], these 29 novel variants are classified as pathogenic or likely pathogenic variants. Notably, the *FUS* p.S235G & p.S236G double mutation identified in a sporadic Chinese bvFTD patient involves two amino acid substitutions within the *FUS* gene. The specific amino acid substitutions are at positions 235 and 236 in the arginine/glycine/glycine (RGG)-rich domain of the *FUS* gene, which resembles the so-called Swedish mutation (K595N/M596L) in *APP* in Alzheimer’s disease.

In cohorts of European ancestry with FTD, the mutation frequencies of *C9orf72*, *GRN*, and *MAPT* in familial cases range from 20–30%, 5–25%, and 5–20% respectively. In sporadic cases, the mutation frequencies are 6% for *C9orf72*, 5% for *GRN*, and 2% for *MAPT *[9]. These three genes show the highest frequencies in FTD. Comparatively, mutations in *TBK1*, *SQSTM1, OPTN,* and *TARDBP* are less frequently reported in Caucasians [37]. However, through meta-analysis, our study indicates *MAPT* mutations are the most common in Chinese FTD patients (3.7%), followed by *TBK1* mutations (2.0%), which occur more frequently than *GRN* (1.7%) and *C9orf72* (0.5%) mutations. These findings reinforce that the genetic spectrum of FTD patients is different between Asians and Caucasians.

Similarly, the frequencies of *TBK1* (2.7%) and *OPTN* (2.3%) pathogenic variants were relatively high in our cohort. In particular, we identified 7 nonsynonymous *TBK1* variants in 261 FTD patients. Six rare missense variants and one frameshift variant were considered probably pathogenic, and all of them were novel. After a systematic review of the literature, eight relevant studies of Chinese cohorts were identified, reporting a total of 12 *TBK1* variants, including one splice variant, eight frameshift variants, and three missense variants. Conversely, *OPTN* variants were less frequently reported, warranting further investigation in this population. The most common frontotemporal lobar degeneration (FTLD) is characterized neuropathologically by the abnormal accumulation of the protein tau (FTLD-tau) in Chinese [10, 38]. However, our study indicates that the abnormal accumulation of the proteins TDP-43 (FTLD-TDP) associated with *TBK1* and *OPTN* genes may be not rare in Chinese populations, and research into the role of these genes contributes to the investigation of key overarching pathways in FTD.

The *TBK1* c.2086G > A p.Glu696Lys variation was previously identified in two independent ALS patients with a similar disease phenotype in Sweden [25]. Both patients presented progressive bulbar palsy with an aggressive course. One of the patients exhibited overt dyscognition and was also diagnosed as having FTD. It was found that the p.Glu696Lys mutation in the TBK1 CCD2 domain inhibited binding to OPTN, indicating haploinsufficiency pathogenesis [25]. Interestingly, OPTN-TBK1 complex formation is significantly enhanced by the TBK1 p.Glu696Gly variant in our results, indicating plausible gain-of-function pathogenesis. Both the *TBK1* p.Glu696Gly and p.Glu696Lys mutations identified in FTD/ALS patients indicated the importance of the E696 residue for TBK1-OPTN interaction in disease pathogenesis.

*TBK1* p.I37T and p.E232Q were novel mutations located in the kinase catalytic domain. Both mutations were associated with aphasia and extrapyramidal symptoms of FTD. Our functional study revealed that the TBK1 I37T mutant decreased OPTN phosphorylation, and TBK1 autophosphorylation was also reduced by both I37T and E232Q mutants. Therefore, loss-of-function mechanisms might be involved in the pathogenesis of FTD caused by these two *OPTN* mutations.

The c.1546G > C p.E516Q variation in *OPTN* was identified in sporadic Chinese ALS patients with rapid disease progressions [39, 40]. Another Chinese p.E516Q mutation carrier presented with typical clinical, electromyographic, and imaging features of ALS-FTD [41]. Patients with *OPTN* p.E516Q mutation reported to date tend to have a rapid progression. The p.L494W mutation in *OPTN* was initially linked to the classic ALS phenotype [41]. Additionally, it was found in a sporadic case of juvenile-onset open-angle glaucoma in a Chinese patient, who had not exhibited any ALS symptoms by the age of 33 [42]. As the mean age at onset of ALS is over the fifties, the patient should be followed at regular intervals. To date, functional studies have not been performed for the two mutants. In our study, both mutants resulted in the formation of vesicular structures that co-localized with LC3 puncta same as OPTN wild-type. Nevertheless, these mutations cannot be necessarily determined as non-pathogenic based on these results alone. Further in-depth functional analyses might be required to validate their pathogenicity.

OPTN coordinates endocytosis and membrane trafficking through its interactions with Rab8 and myosin VI [26]. Unlike the OPTN wild-type, L494W, and E516Q mutants that formed vesicular structures, the OPTN R144G and F475V mutants did not show vesicular localization. These mutants are located in the Rab8 (residues 1–209) or myosin VI (residues 417–512) binding domain, respectively. Previous studies have shown that mutations in these domains impair Rab8-mediated vesicular trafficking [43] and the interaction of myosin VI with optineurin [27]. The reduced binding of Rab8 and myosin VI with the OPTN R144G and F475V mutants may alter the morphology of optineurin from predominately vesicular into a diffuse appearance.

Cells expressing OPTN R144G and F475V mutants displayed decreased recruitment to autophagosomes. During the autophagic processes, optineurin binds to a ubiquitin-decorated cargo and links the ubiquitinated cargo to autophagosomal membranes via binding to LC3 [44]. The OPTN F475V or R144G mutants, located in the UBD or in proximity to the LIR domain—which are the sites for ubiquitinated cargo binding and LC3 binding, respectively—may suppress the recruitment of optineurin to autophagosomes.

This study has several limitations. First, it lacks neuropathological confirmation. Second, further functional validation may be necessary for some of the rare mutations identified in our cohort. Lastly, a larger sample size may be required to accurately assess the frequencies of disease-causing genes in FTD.

In conclusion, we analyzed disease-causing genes in 261 Chinese Han patients with FTD and examined both the clinical and genetic characteristics of patients with rare variants in these genes. Furthermore, we discovered pathogenic mutations in *TBK1* and *OPTN* that were functionally validated, suggesting that *TBK1* and *OPTN* variants might be a common cause of FTD in Chinese. Our findings reinforce the role of autophagic defects and TDP-43 proteinopathy in FTD pathogeneses and will accelerate effective drug development in the future.

### Supplementary Information

Supplementary Material 1: Supplementary Table 1. Genes known to be associated with FTD and other dementia-related neurodegenerative diseases.

Supplementary Material 2: Supplementary Table 2. Distribution of* ApoE*-alleles in our FTD cohort and normal Chinese population.

Supplementary Material 3.

## Data Availability

No datasets were generated or analysed during the current study.
