# Supplementary material for: Genetic and clinical landscape of Chinese frontotemporal dementia: dominance of TBK1 and OPTN mutations
Source: Alzheimers Res Ther. 2024 Jun 13;16:127. doi: 10.1186/s13195-024-01493-w (PMC11170894; doi:10.1186/s13195-024-01493-w)
Supplement: Supplementary file 1 — Supplementary Material 1: Supplementary Table 1. Genes known to be associated with FTD and other dementia-related neurodegenerative diseases. [file 13195_2024_1493_MOESM1_ESM.docx]

Supplementary Table 1. List of genes known to be associated with FTD and other dementia-related neurodegenerative diseases.

| **Phenotype** | **Genes** |
| --- | --- |
| **AD** | *APP, PSEN1, PSEN2* |
| **Associated with or susceptible to AD** | *APOE, A2M, ABCA7, ACE, APBB2, ATXN1, AKT1, AR, BIN1, BLMH, CASP3, CD2AP, CD33, CHCHD10, C9ORF72, CYP2C, CST3, CELF1, CLU, CR1, DNMT1, DSG2, EPHA1, ETS1, FERMT2, GSK3B, GRB2, HTR7, HFE, INPP5D, ITM2B, LRP1, MEF2C, MPO, MS4A4E, MPHOSPH1, MS4A6A, NME8, NOS3, NOTCH3, PICALM, PAXIP1, PLAU, PTK2B, SLC24A4, SORL1, TNF, TREM2, TYROBP, ZCWPW1, CSF1R, AARS2, HTRA1, COL4A1* |
| **FTD** | *BTNL2, C9orf72, CSF1R, CHCHD10, CHMP2B, CST3, CTSC, DCTN1, FUS, GRN, hnRNPA1, hnRNPA2B1, MAPT, OPTN, PRKAR1B, RAB38, SIGMAR1, SOD1, SQSTM1, TBK1, TARDBP, TMEM106B, TREM2, UBQLN2, VCP, CYLD, TIA1, CCNF, TUBA4A, ATXN2, ANXA11, PRNP, CHCHD2, EXT2, EWSR1, TAF15* |
| **Other dementia-related diseases** | *GBA, SNCA, SNCB, ATP13A2, EPM2A, ITM2B, NHLRC1, PRICKLE1, TRPM7, CFAP410, PSAP, EIF4G1, SCN8A, COQ2, TSC1, TSC2, PLA2G6, SLC9A6, LRRK2, PINK1, SERPINI1* |

AD: Alzheimer's disease; FTD: Frontotemporal dementia.
