# Supplementary material for: Genetic and clinical landscape of Chinese frontotemporal dementia: dominance of TBK1 and OPTN mutations
Source: Alzheimers Res Ther. 2024 Jun 13;16:127. doi: 10.1186/s13195-024-01493-w (PMC11170894; doi:10.1186/s13195-024-01493-w)
Supplement: Supplementary file 2 — Supplementary Material 2: Supplementary Table 2. Distribution of ApoE-alleles in our FTD cohort and normal Chinese population. [file 13195_2024_1493_MOESM2_ESM.docx]

Supplementary Table 2. Distribution of *ApoE* alleles in our FTD cohort and Chinese population.

|  | ApoE2 | ApoE3 | ApoE4 |
| --- | --- | --- | --- |
| FTD patients in our cohort (n=261) | 49/522 (9.39%) | 417/522 (79.89%) | 56/522 (10.73%) |
| Chinese control (n=171) [1] | 24/342 (7.02%) | 285/342 (83.33%) | 33/342 (9.65%) |
| p-value | 0.76 | 0.08 | 0.74 |

The variables were compared between the patients in our FTD cohort and the normal Chinese population.

**References**

[1] Seet WT, Mary Anne TJ, Yen TS. Apolipoprotein E genotyping in the Malay, Chinese and Indian ethnic groups in Malaysia-a study on the distribution of the different apoE alleles and genotypes. Clin Chim Acta. 2004;340:201-5.
