# Supplementary figures and images for: Genetic and clinical landscape of Chinese frontotemporal dementia: dominance of TBK1 and OPTN mutations
Source: Alzheimers Res Ther. 2024 Jun 13;16:127. doi: 10.1186/s13195-024-01493-w (PMC11170894; doi:10.1186/s13195-024-01493-w)

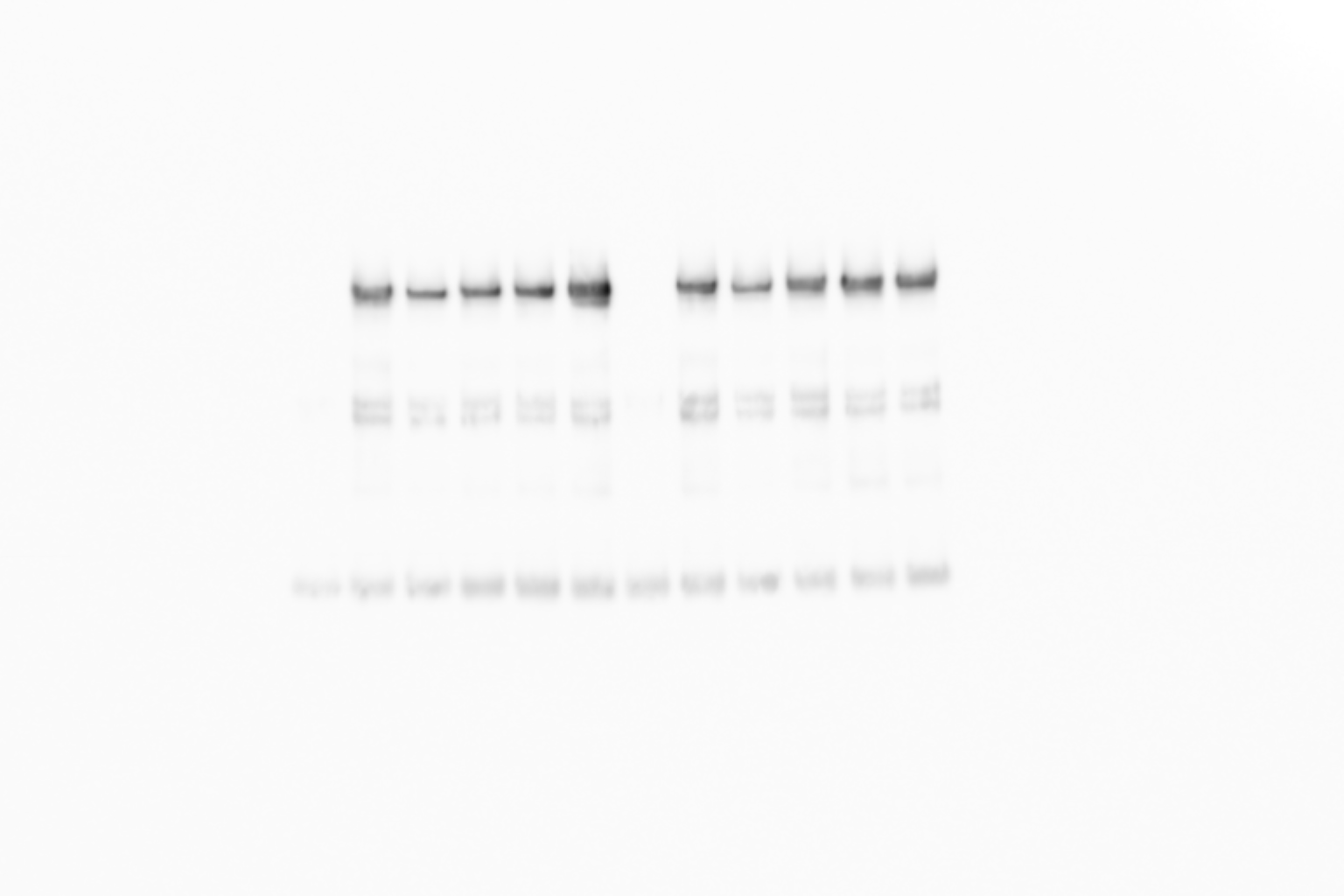

Supplement: Supplementary file 3 — Supplementary Material 3. [file 13195_2024_1493_MOESM3_ESM.zip › WB-Data/FIPFlagtbk1_7.tif]

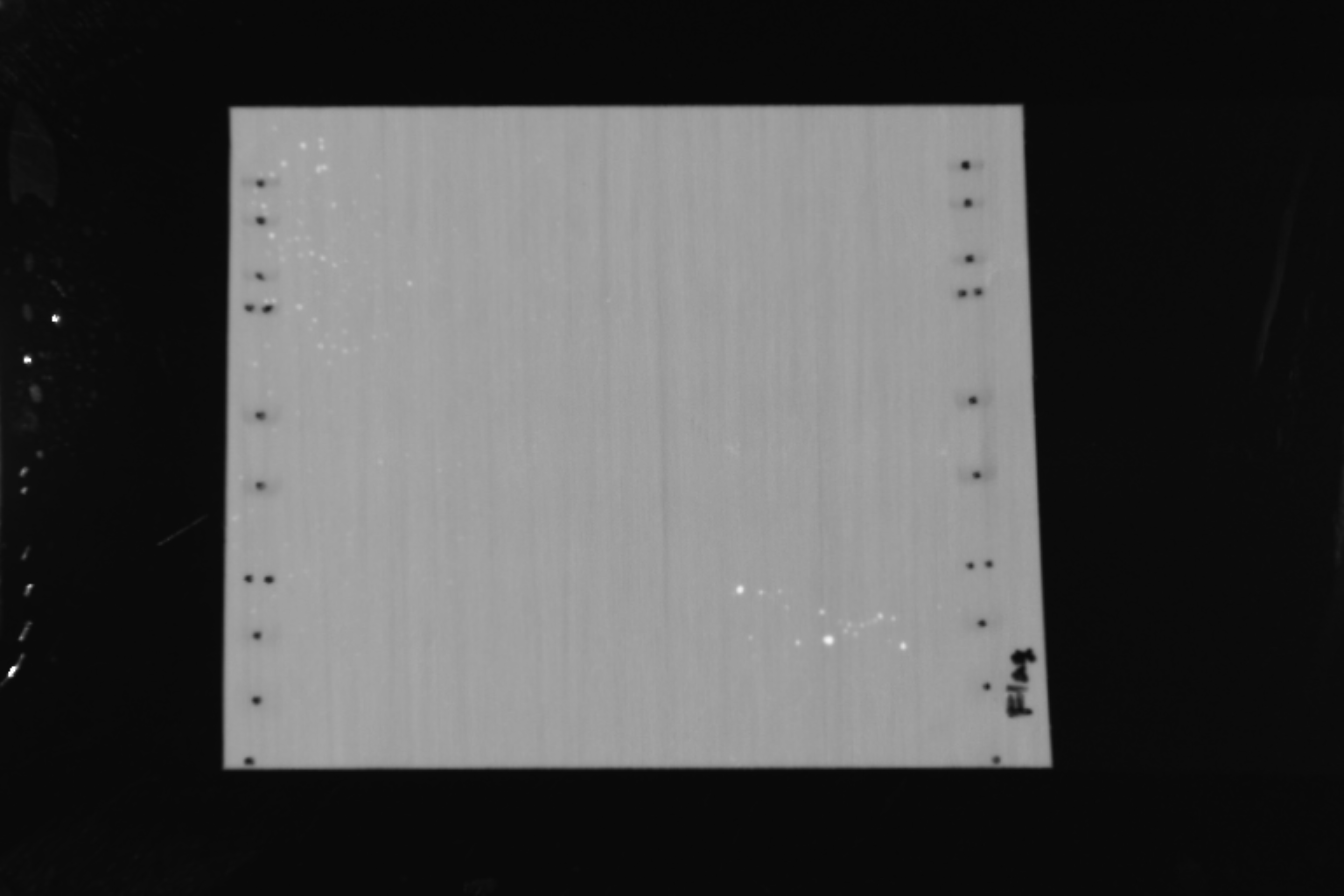

Supplement: Supplementary file 3 — Supplementary Material 3. [file 13195_2024_1493_MOESM3_ESM.zip › WB-Data/FIPFlagtbk1_mk.tif]

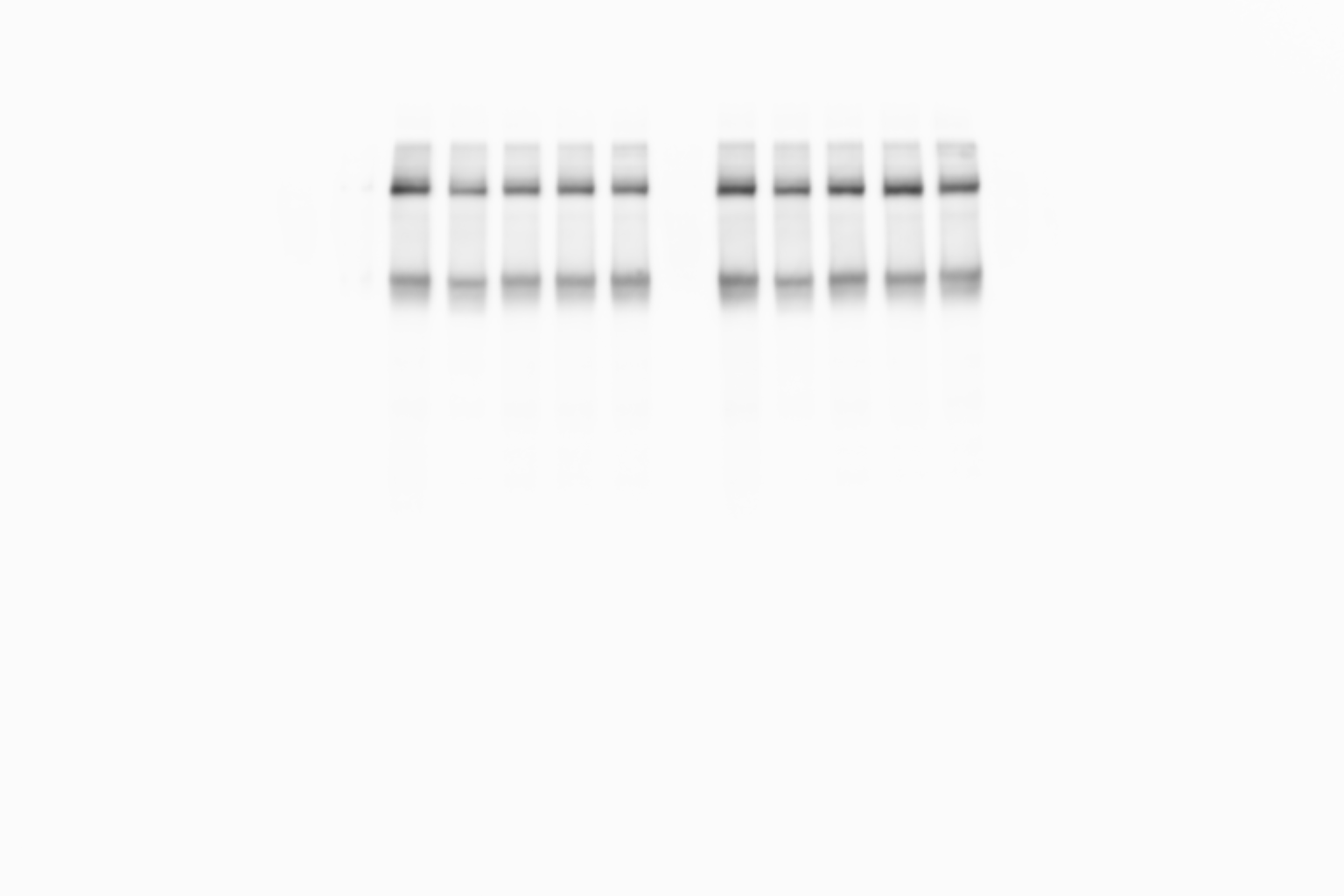

Supplement: Supplementary file 3 — Supplementary Material 3. [file 13195_2024_1493_MOESM3_ESM.zip › WB-Data/Flag-TBK1_1.tif]

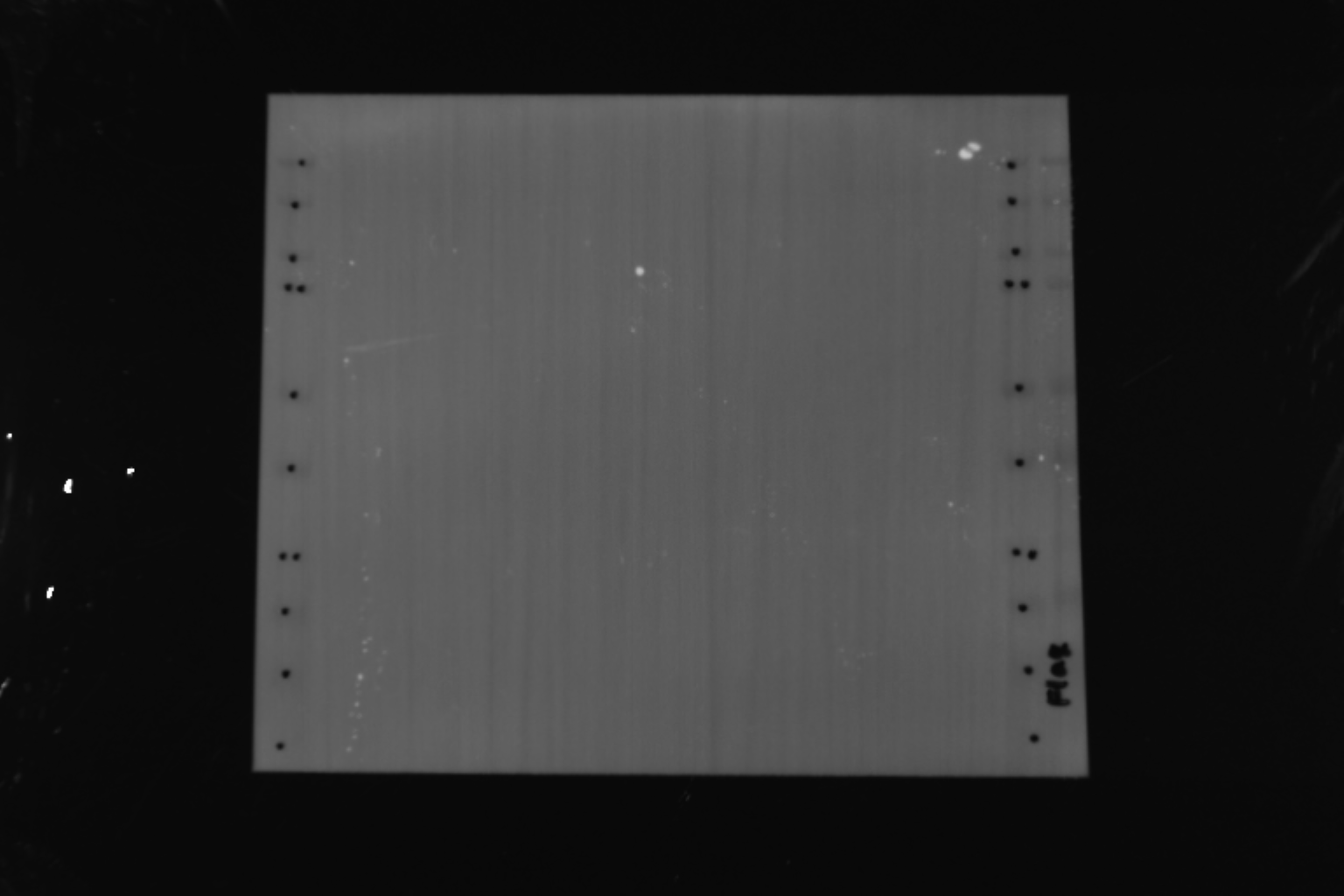

Supplement: Supplementary file 3 — Supplementary Material 3. [file 13195_2024_1493_MOESM3_ESM.zip › WB-Data/Flag-TBK1_mk.tif]

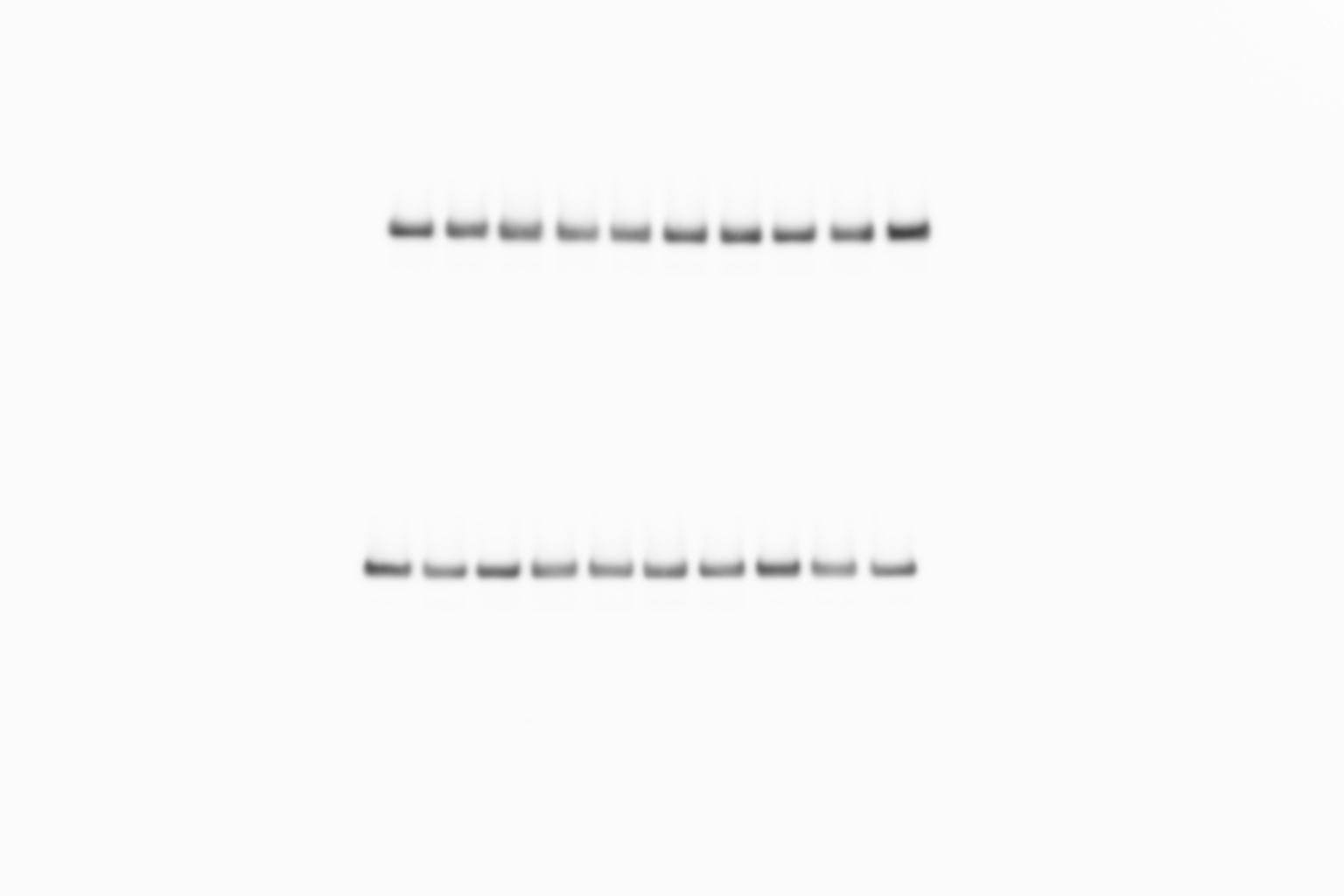

Supplement: Supplementary file 3 — Supplementary Material 3. [file 13195_2024_1493_MOESM3_ESM.zip › WB-Data/GAPDH_2.tif]

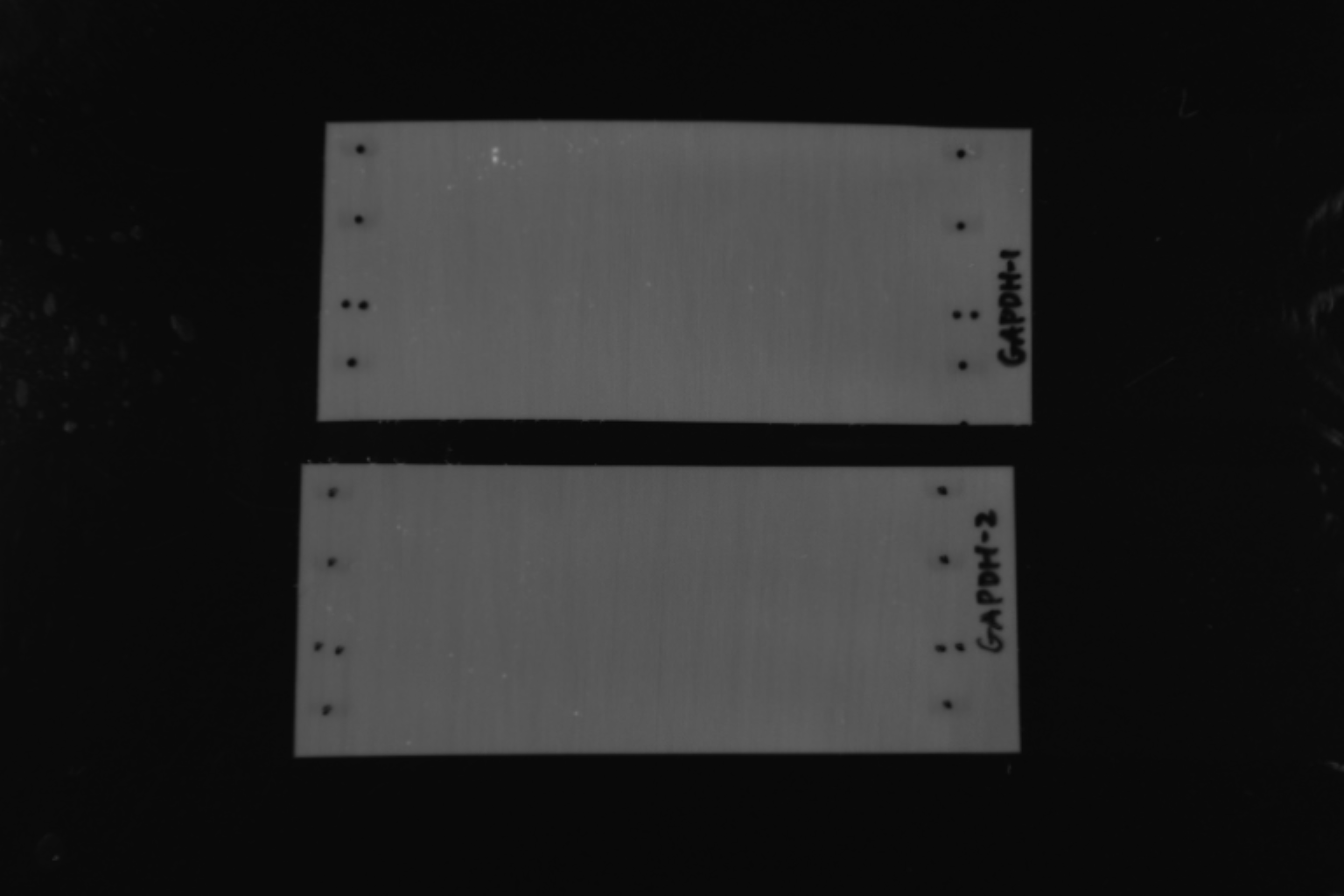

Supplement: Supplementary file 3 — Supplementary Material 3. [file 13195_2024_1493_MOESM3_ESM.zip › WB-Data/GAPDH_mk.tif]

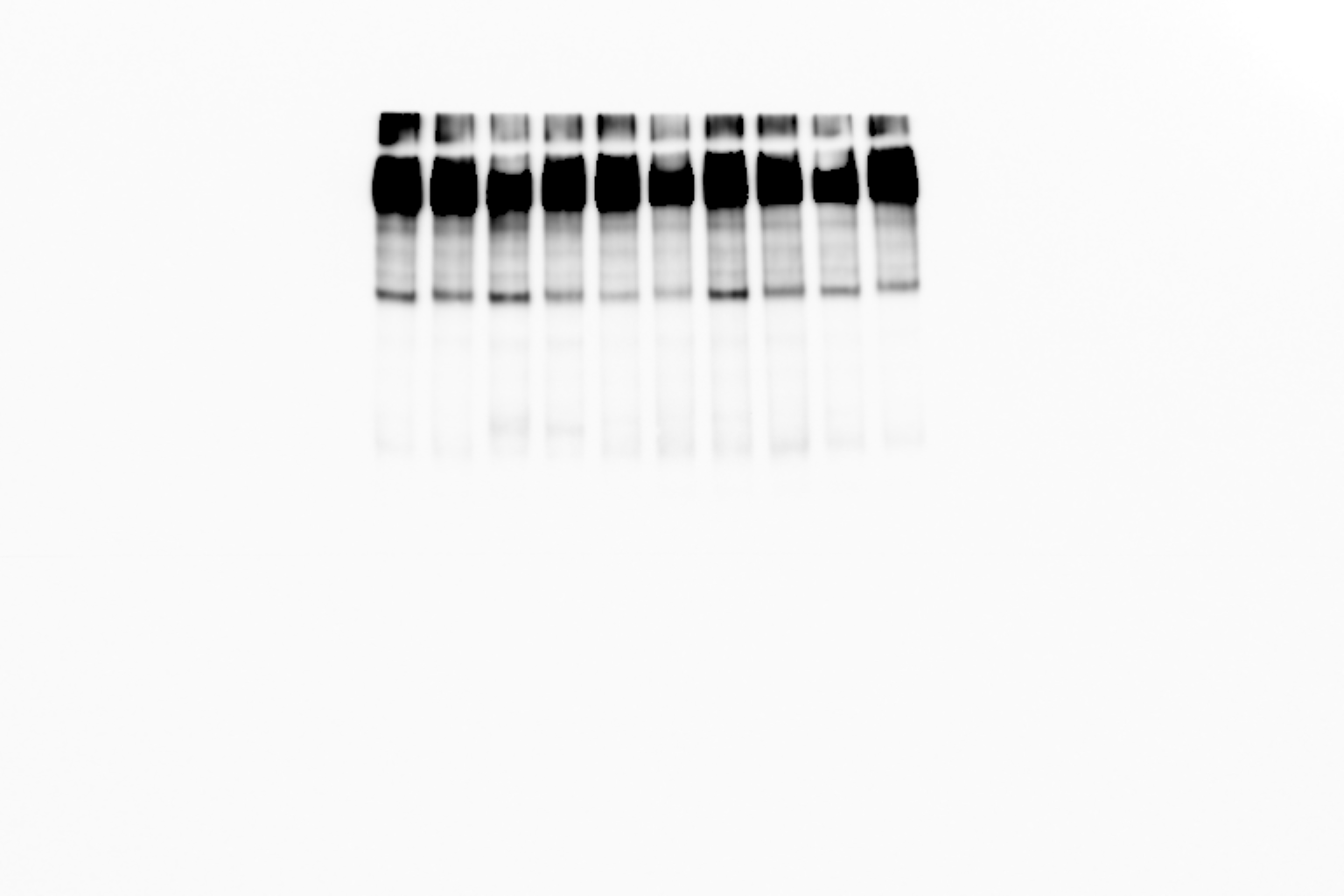

Supplement: Supplementary file 3 — Supplementary Material 3. [file 13195_2024_1493_MOESM3_ESM.zip › WB-Data/mCh_OPTN_High_11.tif]

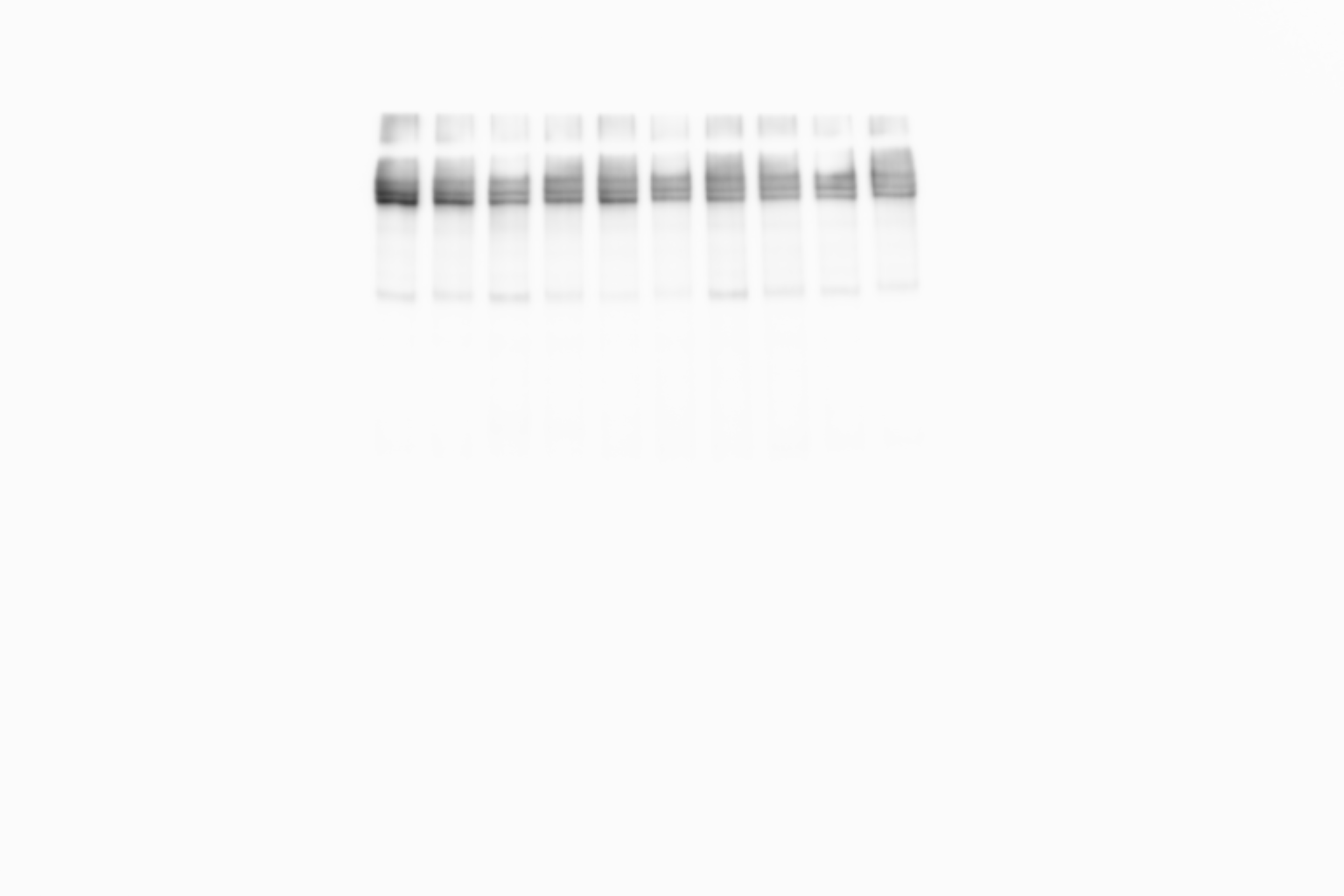

Supplement: Supplementary file 3 — Supplementary Material 3. [file 13195_2024_1493_MOESM3_ESM.zip › WB-Data/mCh_OPTN_Low_1.tif]

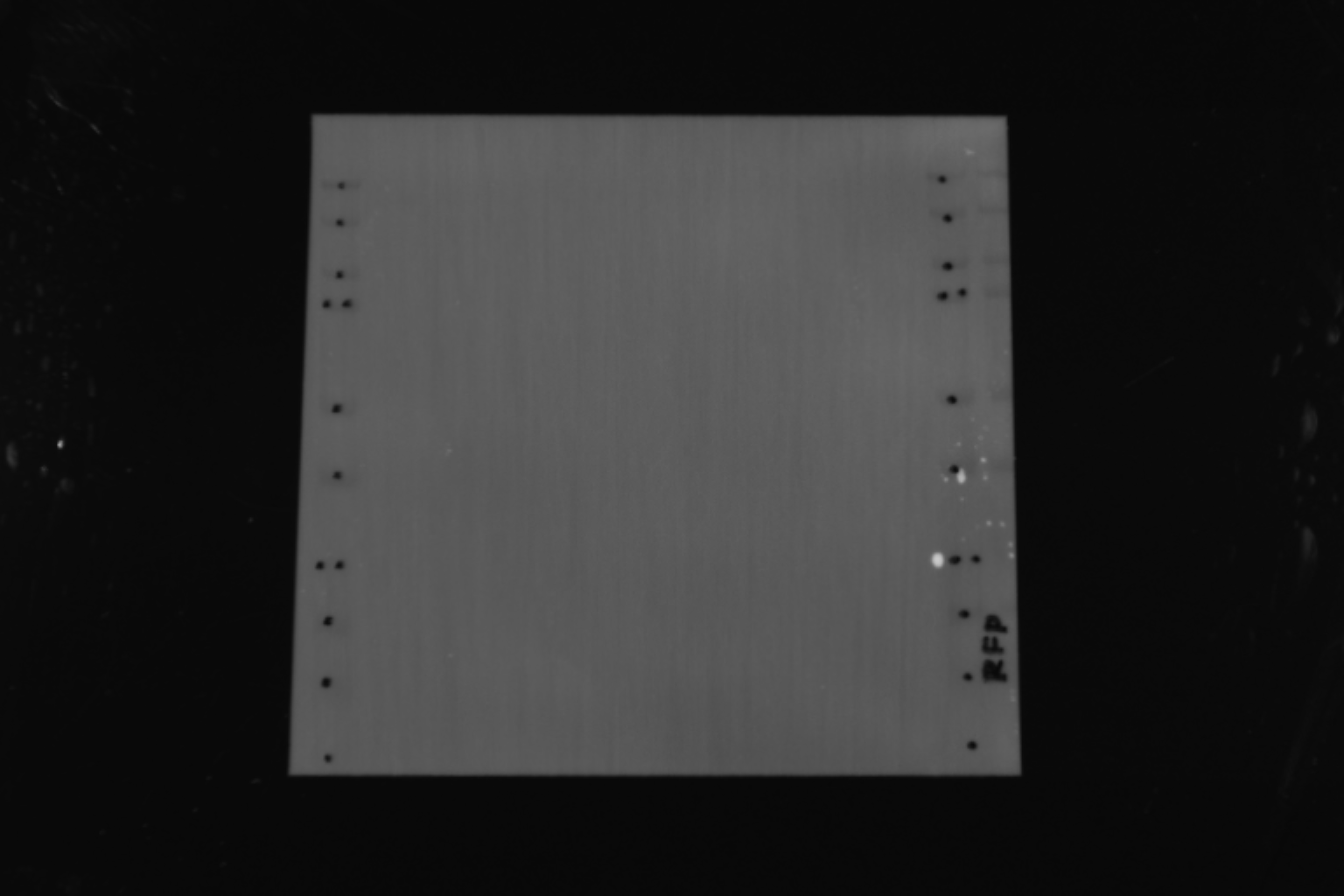

Supplement: Supplementary file 3 — Supplementary Material 3. [file 13195_2024_1493_MOESM3_ESM.zip › WB-Data/mCh_OPTN_mk.tif]

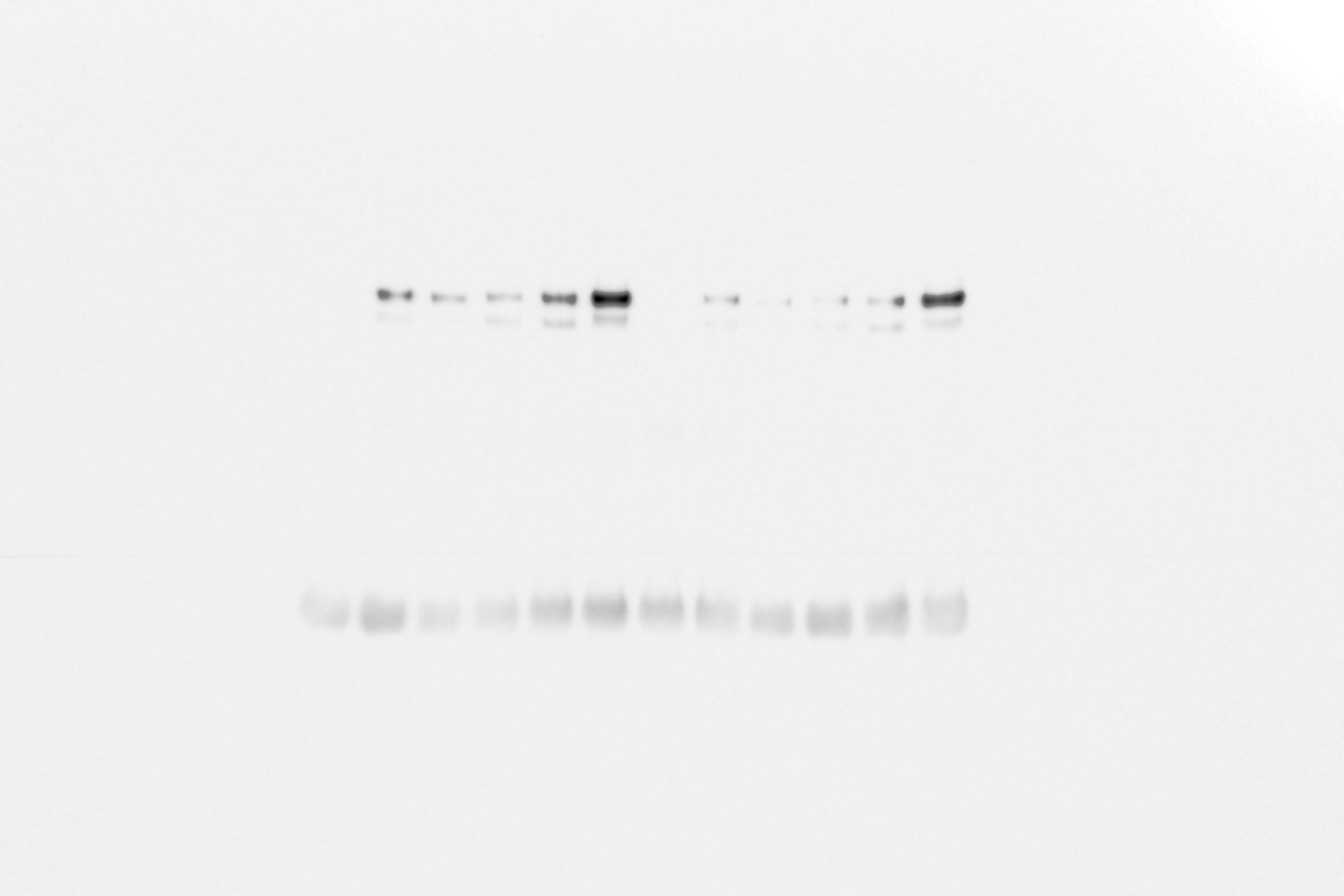

Supplement: Supplementary file 3 — Supplementary Material 3. [file 13195_2024_1493_MOESM3_ESM.zip › WB-Data/mch-OPTN_10.tif]

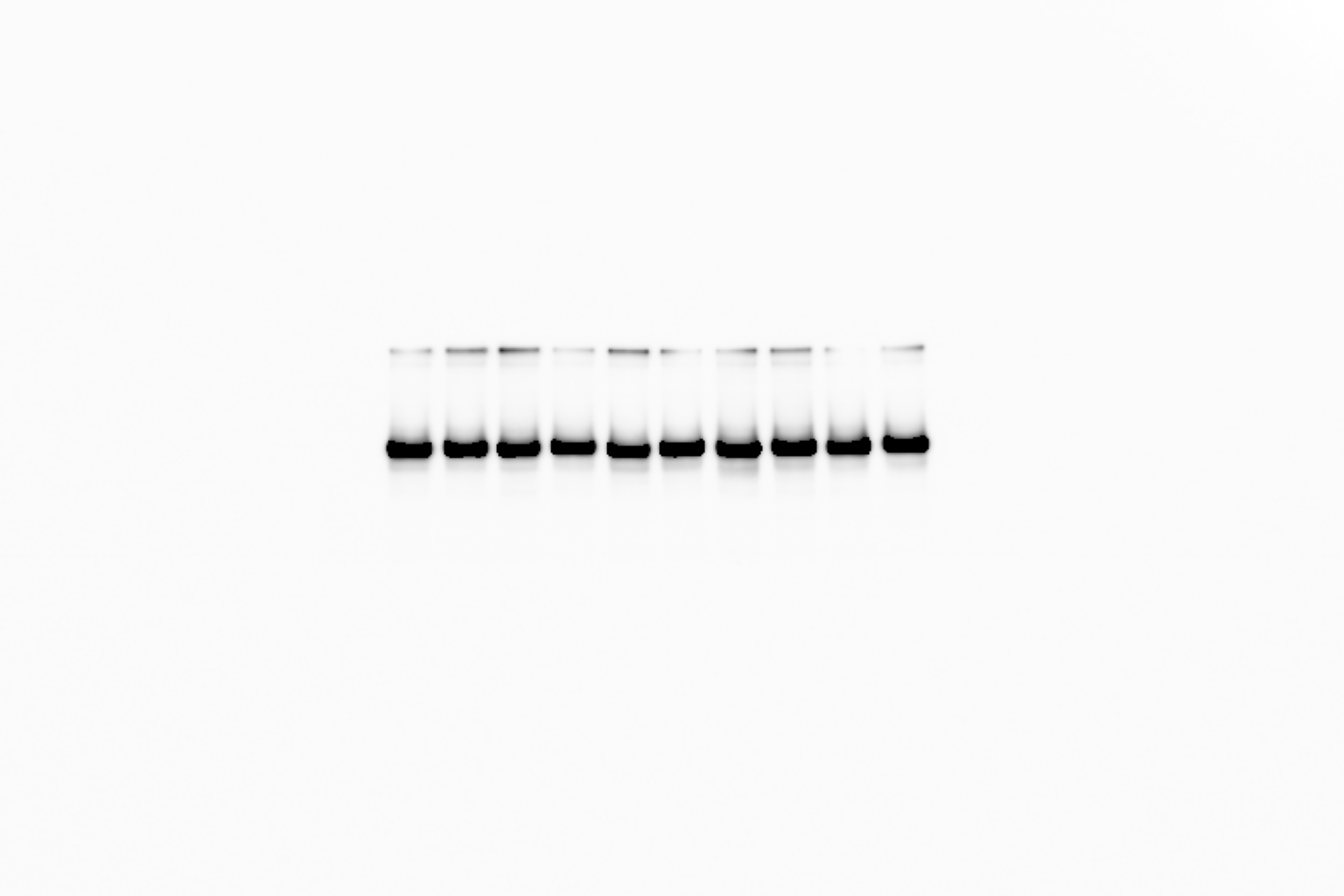

Supplement: Supplementary file 3 — Supplementary Material 3. [file 13195_2024_1493_MOESM3_ESM.zip › WB-Data/mCh-OPTN_High_5.tif]

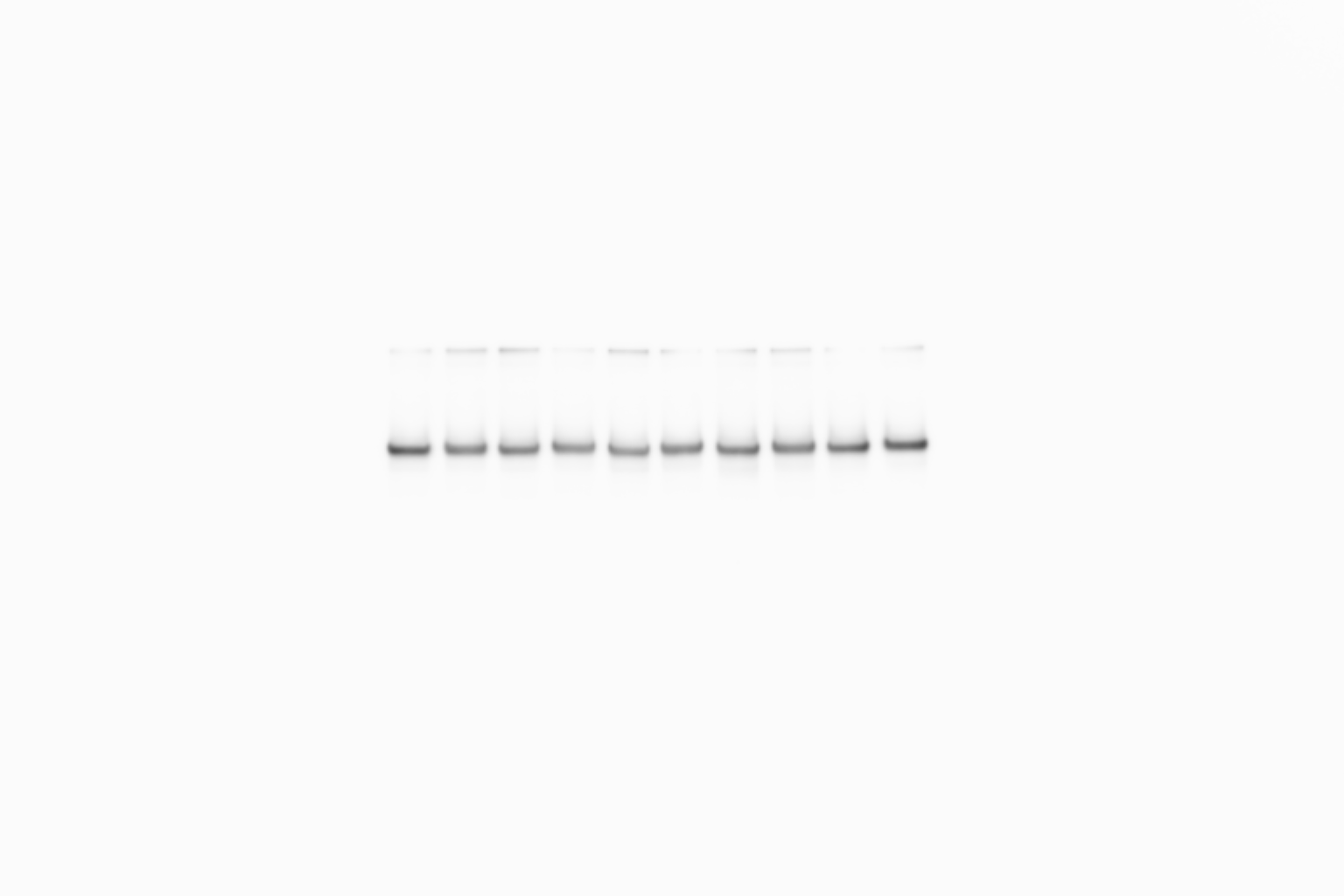

Supplement: Supplementary file 3 — Supplementary Material 3. [file 13195_2024_1493_MOESM3_ESM.zip › WB-Data/mCh-OPTN_Low_1.tif]

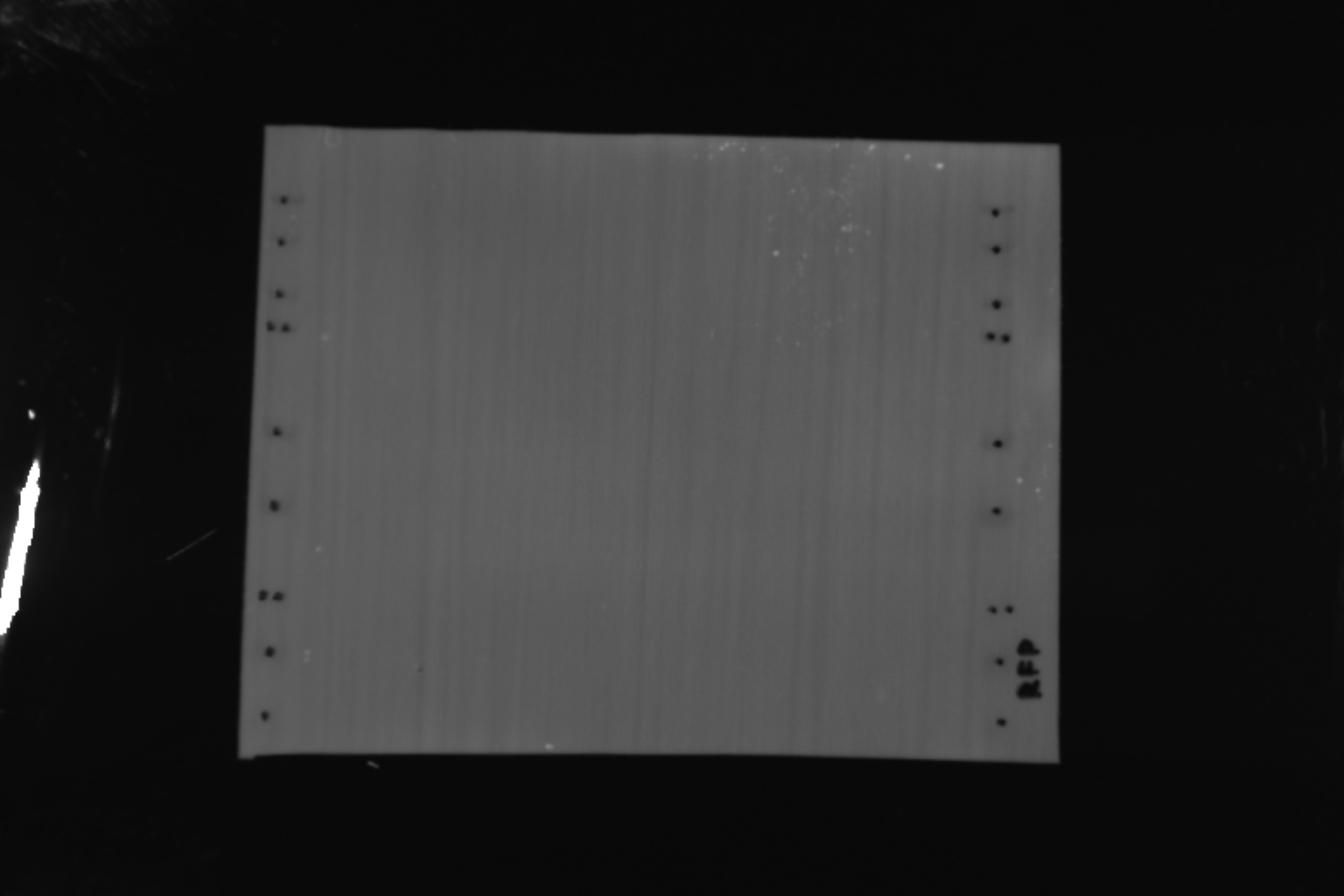

Supplement: Supplementary file 3 — Supplementary Material 3. [file 13195_2024_1493_MOESM3_ESM.zip › WB-Data/mch-OPTN_mk.tif]

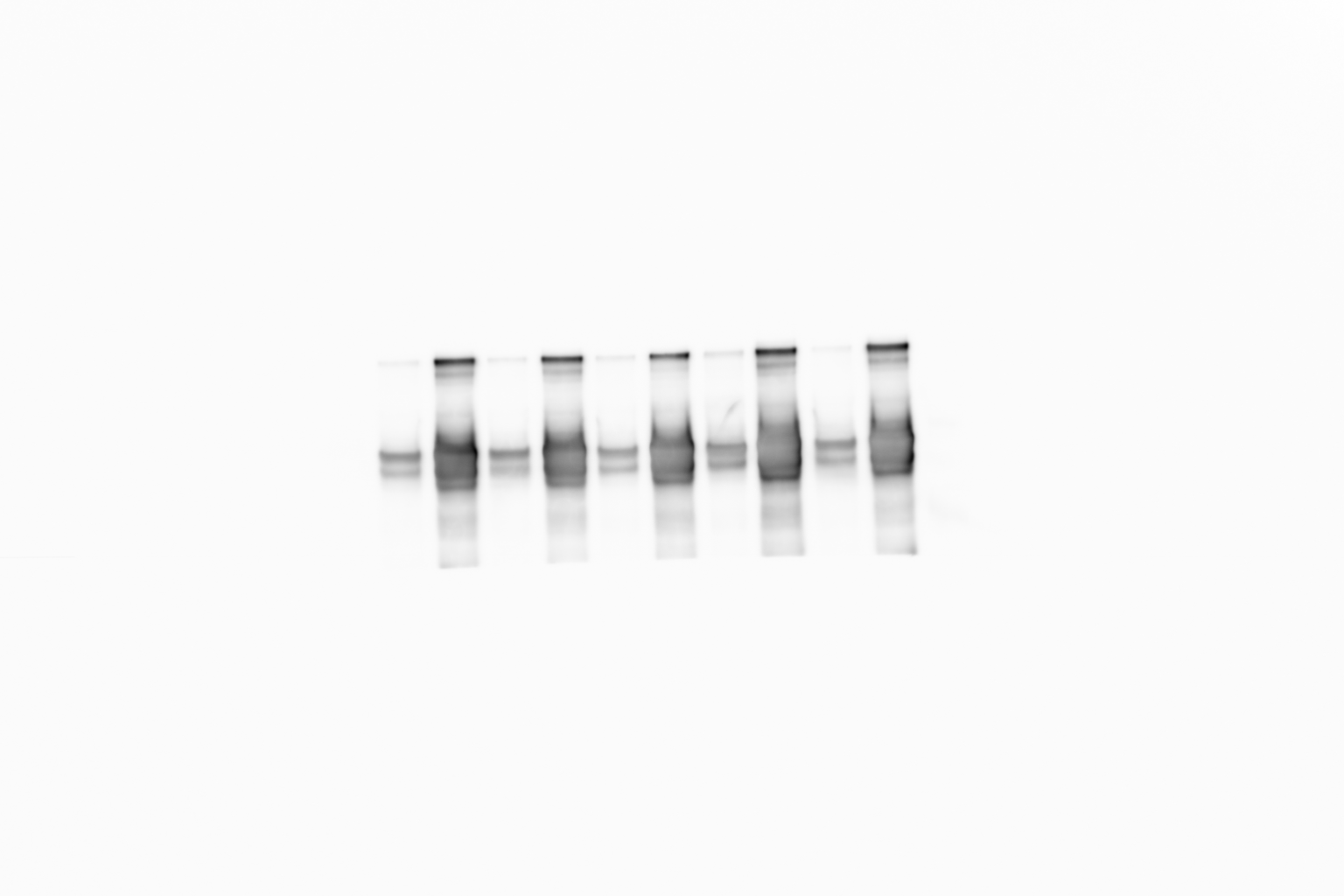

Supplement: Supplementary file 3 — Supplementary Material 3. [file 13195_2024_1493_MOESM3_ESM.zip › WB-Data/pOPTN(pS177)_11.tif]

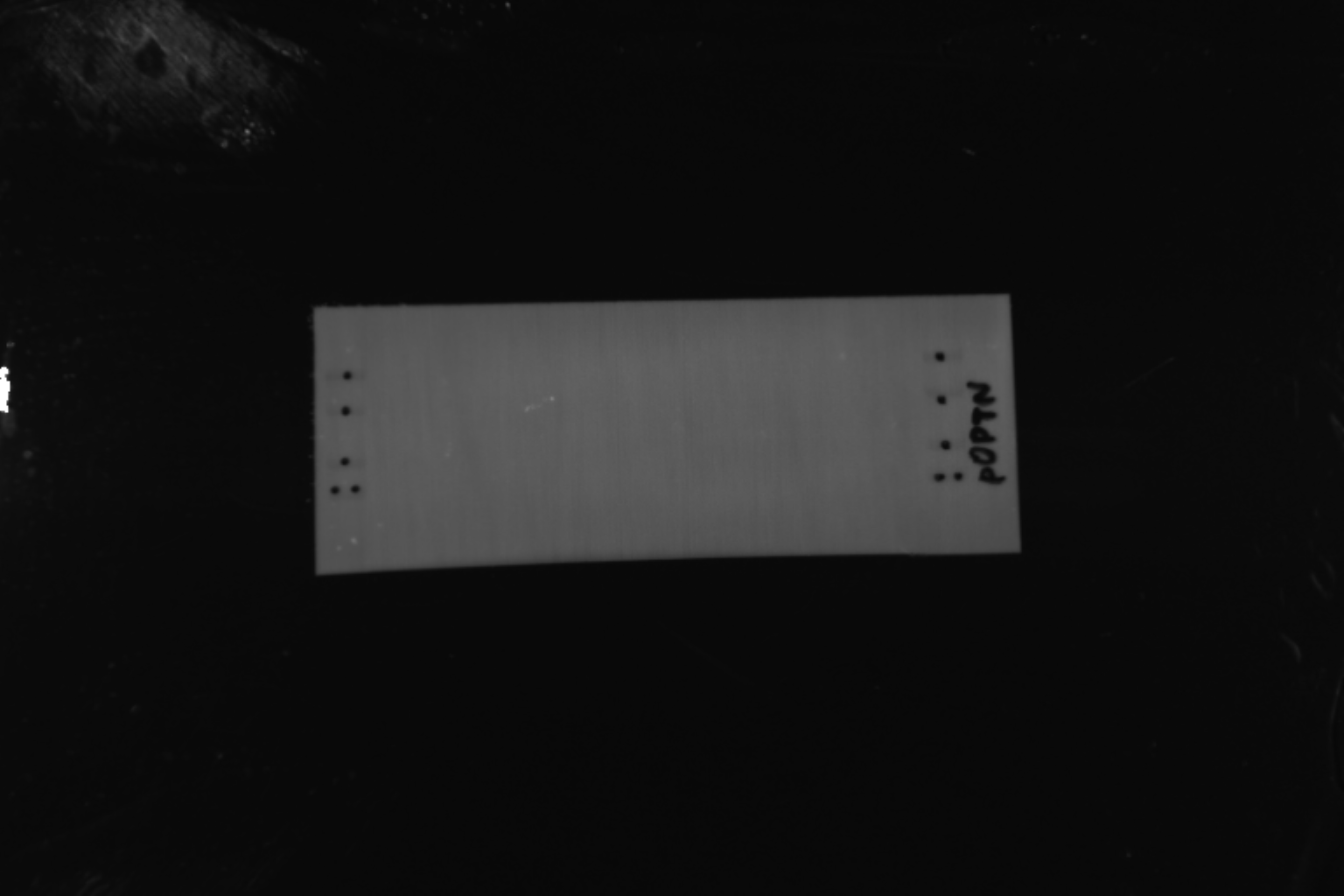

Supplement: Supplementary file 3 — Supplementary Material 3. [file 13195_2024_1493_MOESM3_ESM.zip › WB-Data/pOPTN(pS177)_mk.tif]
